# Evaluation and identification of advanced inter-specific derivatives from crosses of *Cicer arietinum* with *C. reticulatum* and *C. echinospermum* for agro-morphological, quality traits and disease resistance

**DOI:** 10.3389/fpls.2024.1461280

**Published:** 2024-09-27

**Authors:** Amool Singh Vadithya, Inderjit Singh, Upasana Rani, Sreya Venadan, Rajdeep Jajoriya, Mohar Singh, Harpreet Kaur Oberoi, Sarvjeet Singh, Chellapilla Bharadwaj, Shayla Bindra

**Affiliations:** ^1^ Department of Plant Breeding and Genetics, Punjab Agricultural University, Ludhiana, India; ^2^ ICAR-National Bureau of Plant Genetic Resources Regional Station, Shimla, India; ^3^ Division of Genetics, ICAR- Indian Agricultural Research Institute, New Delhi, India

**Keywords:** Inter-specific hybridization, variability parameters, correlations, path analysis, principal component analysis, biotic stresses, crude protein

## Abstract

Inter-specific hybridization is a key strategy in modern crop improvement, aiming to integrate desirable traits from wild species into cultivated backgrounds. This study delves into the evaluation and identification of advanced inter-specific derivatives (IDs) derived from crosses of cultivated chickpea with *Cicer reticulatum* and *C. echinospermum*. The primary aim was to incorporate desirable yield enhancement traits, disease resistance, and nutritional quality traits into cultivated chickpea. The IDs were assessed during *rabi* 2021-22 and 2022-23 in the northern plains zone of India. Significant amount of genetic variability was observed for key agro-morphological traits having high heritability and genetic advance. Superior derivatives were identified for early flowering, high seed yield, and resistance to Ascochyta blight, Botrytis grey mould, and Fusarium wilt. Significant variability for crude protein and total soluble sugar content was also observed among the derivatives. The findings highlight the potential of utilizing wild *Cicer* species to broaden the genetic base of cultivated chickpea for the development of robust, high-yielding, disease-resistant varieties with improved nutritional traits suitable for diverse environmental conditions. The superior derivatives identified in this study hold promise for future breeding programmes for improving productivity, disease resistance and nutritional quality.

## 1 Introduction

Chickpea (*C. arietinum* L.) is a temperate, autogamous legume with a chromosome number of 2n = 2x = 16 and a haploid genome consisting of 738 mega bases (Varshney et al., 2012). It holds the second position globally among food legumes after beans. *C. arietinum* is the only cultivated species of genus *Cicer* including 10 annual and 36 perennial species (Toker et al., 2021). Its wild ancestor is believed to be *C. reticulatum*. The historical roots of chickpea dates back to 7,500-6,800 BC, with archaeological findings locating its origin in the Middle East, particularly in southeastern Turkey and adjoining parts of Syria (van der Maesen, 1987; Zohary et al., 2012). This legume is regarded as a nutritional powerhouse, offering proteins, vitamins (such as niacin and thiamine), minerals, carbohydrates and essential unsaturated fatty acids (linolenic and oleic acids) (Heiras-Palazuelos et al., 2013; Singh et al., 2022a). It holds a significant place in diets, especially for those unable to access animal protein or adhering to vegetarianism in semi-arid regions. Moreover, combining pulses like chickpea with cereals ensures a balanced intake of essential amino acids to complement each other’s deficiencies (Reinkensmeier et al., 2015).

Chickpea is cultivated on approximately 14.81 million hectares globally, yielding a total production of 18.09 million tonnes with an average productivity of 1221.8 kg/ha. India remains the largest producer and consumer of chickpea, contributing around 75 per cent (13.56 million tonnes) of the global production, with 10.74 million hectares under cultivation in 2022 (FAOSTAT, 2022). To achieve self-sufficiency in pulse production by 2050 (as per Vision 2050 document of IIPR, Kanpur), India needs to reach a total pulse production of 39 MT with chickpea production targets of about 16-17.5 MT from an area of 10.5 million hectares (Dixit et al., 2019). Notably, the absence of genetically improved crop varieties and limited genetic diversity leads to significant breeding challenges. This is primarily due to the origin of chickpeas from a single domestication event followed by high rates of self-pollination (Abbo et al., 2003, 2005; Chaturvedi and Nadarajan, 2010). To address the issue of limited genetic diversity, emphasis has been directed towards harnessing traits from crop wild relatives (CWRs) for yield enhancement and stress resistance (Croser et al., 2003; Mallikarjuna et al., 2007; Bains et al., 2012). Studies on wild *Cicer* species on variability for essential traits and potential compatibility with cultivated chickpea highlight the importance of broadening the genetic base of alien introgression (Xiao et al., 1996; Tanksley and McCouch, 1996). Consequently, in pursuit of additional achievements to enhance crop yield and consistency in forthcoming plant varieties, it is necessary to integrate novel desirable traits into the existing cultivated chickpea background. Furthermore, enriching the cultivated gene pool with complementary genes and alleles from CWRs is essential for maximizing genetic gains by selection (Vega and Frey, 1980).

Inter-specific hybridization is essential for crop improvement to introduce novel genetic variation from wild relatives into cultivated species. In chickpea breeding, inter-specific hybridization, involves crossing chickpeas with wild relatives to generate new genetic variation. For example, genes from *C. echinospermum* and *C. reticulatum* have been introgressed into cultivated chickpeas, resulting into significant yield improvements and enhanced tolerance to drought and heat (Singh and Ocampo, 1997; Canci and Toker, 2009; Singh et al., 2024). Similarly, in pigeonpea, inter-specific hybridization with wild relatives such as *Cajanus scarabaeoides* has been used to introgress traits like increased productivity and resistance to pests and diseases, including pod borer and Phytophthora stem blight (Singh et al., 2020).

Various stresses affecting yield and stability are the major restraining factors for the expression of chickpea’s genetic potential. The crop faces significant challenges from various well-documented pathogens (Nene and Reddy, 1987). Among these, productivity is suffered notably by diseases like Ascochyta blight (AB), Botrytis grey mould (BGM) and Fusarium wilt (FW). Ascochyta blight caused by *Ascochyta rabiei*, spreads via seed and crop residue, inflicting severe symptoms like stem breakage, twig collapse, and pod infection (Sharma and Muehlbauer, 2007). Moreover, the pathogen’s evolving nature continually disrupts the resistance mechanisms in newly bred chickpea varieties (Nene and Reddy, 1987; Chen et al., 2004; Kanouni et al., 2011). Similarly, in BGM (causal organism: *Botrytis cinerea* Pers. ex. Fr.), symptoms are; white colonies on stems, leaves, and twigs, with water-soaked lesions (Thakur et al., 2023). Its diverse mechanisms of infection and ability to survive in different forms make the management of BGM in agricultural settings immensely challenging (Brandhoff et al., 2017). Besides, the FW caused by *Fusarium oxysporum* f. sp. *ciceris* results in significant annual yield losses ranging from 10–30% and often a complete crop loss in wilt-sick areas (Sunkad et al., 2019). Although seed dressing and foliar fungicides help manage these diseases, yet the approach is unsustainable, uneconomical, and environmentally hazardous. Under such conditions, host-plant resistance is the most effective and sustainable solution (Pande et al., 2007). The lack of stable resistance in the cultivated gene pool, warrants the need to introgress resistance from CWRs. The resistant sources among wild species like *C. reticulatum* and *C. echinospermum* from the primary gene pool of chickpea possess novel genes for resistance to AB and BGM (Singh et al., 1991; Ramgopal, 2006; Kaur et al., 2013) and FW (Sharma and Muehlbauer, 2007; Mallikarjuna et al., 2011). Moreover, accessions of *C. judaicum*, *C. pinnatifidum*, and *C. bijugum* from the secondary/tertiary gene pools display resistance but are inaccessible for chickpea breeding due to various crossability barriers (Mallikarjuna et al., 2011). Despite the challenges of wide crossing, the successful gene transfer from these wild relatives can improve chickpea disease resistance (Collard et al., 2003; Mallikarjuna et al., 2011). Hence, this study aims to assess and identify superior high-yielding IDs that withstand major biotic challenges and display better nutritional quality for use in developing chickpea cultivars with improved traits.

## 2 Materials and methods

### 2.1 Plant materials, experimental site and design

**Figure 1:**
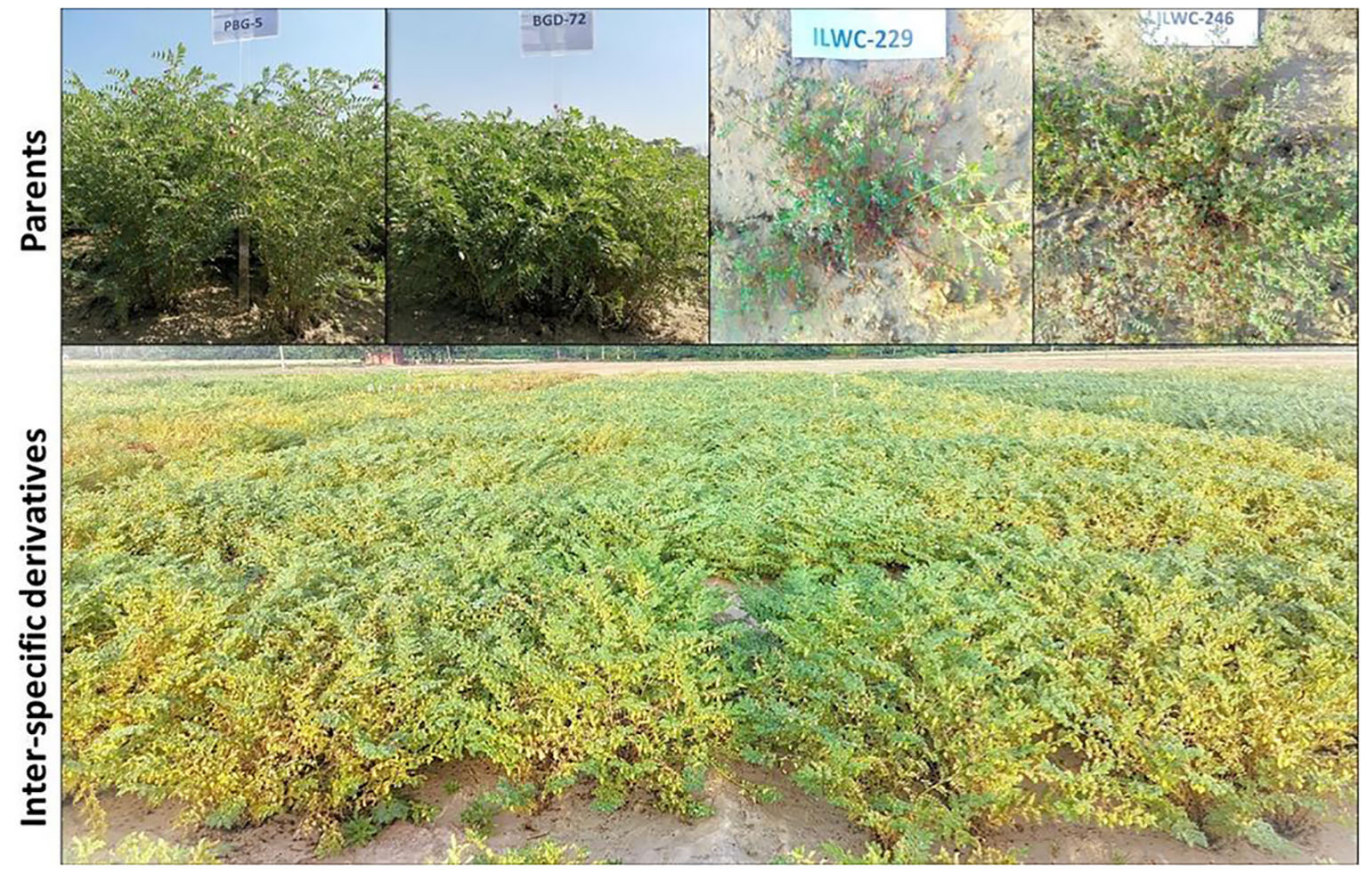
Field view of parents and inter-specific derivatives in field trials at PAU, Ludhiana.

In this study, advanced chickpea IDs derived from crosses between the cultivated chickpea varieties PBG5 and BGD72 (*C. arietinum*) with the wild annual *Cicer* species ILWC229 (EC720438) (*C. reticulatum*) and ILWC246 (EC720481) (*C. echinospermum*) were used (Singh et al., 2018). The segregating populations were advanced using the single seed descent (SSD) breeding technique (Goulden, 1939) to generate derivatives. Finally, a set of 90 IDs from four different crosses namely PBG5 × ILWC229 (33 IDs), BGD72 × ILWC229 (30 IDs), PBG5 × ILWC246 (8 IDs), and BGD72 × ILWC246 (19 IDs) were evaluated for agro-morphological and nutritional traits and against major diseases. A total of 96 genotypes including 90 IDs, their four parents (**Figure 1**) and two standard checks (PBG7 and PBG8) were planted in paired rows of 2-meter length with a row-to-row spacing of 30 cm in alpha lattice design having 8 blocks with two replications during the *rabi* season of 2021-2022 and 2022-2023 at the experimental area of Punjab Agricultural University (PAU), Ludhiana (30° 54′N, 75° 48′E) of Punjab, India. The experimental area falls in the North Western Plains Zone (NWPZ) of India with semi-arid climate having loamy sand soil with a pH range of 7.8-8.2. The average annual rainfall received at Ludhiana is 726 mm. During the 2021-22 *rabi* season, Ludhiana experienced 111.20 mm of rainfall during the crop period, with temperatures ranging from a minimum of 11.19°C to a maximum of 24.84°C. In the subsequent 2022-23 *rabi* season, the region saw an increase in rainfall to 126.70 mm, while the temperature slightly varied, with minimum and maximum values of 11.11°C and 24.26°C, respectively.

### 2.2 Observations of agro-morphological traits

The agro-morphological traits assessed in the study consisted of days to 50% flowering (DFF), days to maturity (DTM), plant height (PH), primary branches per plant (PBPP), secondary branches per plant (SBPP), number of pods per plant (NPP), seeds per 10 pods (SPTP), 100-seed weight (HSW), seed yield per plot (SYPP), biological yield per plot (BYPP) and harvest index (HI). To measure the PH, NPP, PBPP, SBPP and HSW, five plants were randomly taken from each plot, while the traits namely DFF, DTM, SYPP, BYPP and HI were recorded on plot basis.

### 2.3 Disease screening of inter-specific derivatives

#### 2.3.1 Ascochyta blight (*A. rabiei*)

For screening against AB, all the derivatives from four inter-specific crosses along with their parents and highly susceptible checks (L550 and C214) were planted in 2-meter row lengths with a row-to-row spacing of 40 cm during the *rabi* season of 2021-2022 and 2022-2023 in the Ascochyta screening plot, PAU, Ludhiana, India. All plants of the test entries were inoculated by spraying with a conidial suspension of *A. rabiei* (4 × 10^4^ spores ml^-1^) in the evening during the first week of February at the time of flowering and pod initiation (85 to 90 days after sowing) to establish uniform disease for screening. Water was sprinkled from the day following inoculation for 10 min at two-hour intervals to maintain >85% relative humidity for 21 days using perfo-spray system. Disease symptoms started to appear around 10-15 days after inoculation and the observations were made on the response of plants at both vegetative and reproductive stages, using a rating scale from 1 to 9 (Pande et al., 2010). During disease screening, the IDs were sorted into different classes based on their response to the pathogen as ‘1’ for asymptomatic (HR), ‘3’ resistant (R), ‘5’ moderately resistant (MR), ‘7’ susceptible (S), and ‘9’ highly susceptible (HS).

#### 2.3.2 Botrytis grey mould (*B. cinerea*)

All the derivatives from four inter-specific crosses along with their parents and highly susceptible checks (BG1053 and GPF2) were screened using the cut-twig method under laboratory conditions during the *rabi* of 2021-2022 and 2022-23. In this screening experiment, each derivative was replicated twice. A twig from a young plant was collected and briefly immersed in water. After removing it from the water, the cut sections of the plant were treated with rooting hormone before planting them in trays. After planting, the trays were kept in shade and watered twice daily until the roots began to emerge. Thereafter, these trays were transferred to growth chambers, watered, and inoculated with BGM (*B. cinerea*) spore suspension (2 × 10^4^ spores ml^-1^). Following inoculation, the growth chambers were wrapped with polythene sheets to maintain regulated conditions (Temperature: 20°C, Relative Humidity: >90%, and alternating dark and light intervals of 8 and 16 hours, respectively). Disease symptoms were started to appear 3-4 days following inoculation and observations were recorded on sixth-day using 1 to 9 disease rating scale (Pande et al., 2006), where ‘1’ is asymptomatic (HR), ‘3’ resistant (R), ‘5’ moderately resistant (MR), ‘7’ susceptible (S), and ‘9’ highly susceptible (HS).

#### 2.3.3 Fusarium wilt (*F. oxysporum f.sp. ciceris*)

For screening against FW, all the IDs along with their parents and highly susceptible check, JG62, were planted in 4-meter length row with a row-to-row spacing of 40 cm during *rabi* 2021-22 and 2022-23 in the wilt-sick plot, PAU, Ludhiana, India. The wilt-sick plot is well-established with *F. oxysporum f.sp. ciceris* inoculum. After one month of planting, the disease started appearing. A total of three assessments were conducted throughout the crop season to calculate the percentage of plant mortality based on the total number of plants germinated and the number of plants killed by wilt. The categorization of genotypes based on disease incidence was done as per Haware et al. (1992), where asymptomatic (HR) had 0% plant mortality, 0.1-10.0% for resistant (R), 10.1-25.0% for moderately resistant (MR), 25.1-50.0% for susceptible (S) and >50% for highly susceptible (HS).

### 2.4 Extraction and determination of crude protein content and total soluble sugars

The crude protein content was determined using the Kjeldahl method as described by McKenzie and Wallace (1954). Initially, 0.1 g of seed powder obtained by grinding the seeds was wrapped in Whatman No. 1 filter paper and placed in a Kjeldahl digestion tube. A digestion mixture containing copper sulphate and potassium sulphate in 1:9 ratio was added, followed by the addition of 10 ml of concentrated sulphuric acid. After digestion, distillation was conducted using KELPLUS and the resulting samples were titrated with 0.1 N HCL. The determined nitrogen content was converted to protein content using a conversion factor of 6.25.

For extracting total sugars, the method described by AOAC (1965) was employed. The subsequent analysis was done using the phenol sulphuric acid technique developed by Dubois et al. (1956). The sugars were extracted twice with 80 and 70 per cent ethanol by retaining the tubes connected with water condensers in the hot water bath. After each extraction, the supernatants were collected, and 2 ml of the extract was combined with distilled water in a test tube. Following this, 5 per cent phenol and 95.5 per cent sulphuric acid were added, and the absorbance of the resulting pink colour was measured at 490 nm using a spectrophotometer.

### 2.5 Statistical analysis

Statistical analysis was performed for all the traits recorded, wherein mean values from two replications were used. The analysis of variance (ANOVA) for alpha lattice design was done using the R software (version 4.2.1) “agricolae package” and “PBIB.test” functionality. The descriptive statistics was performed on the numerical data using MS Office Excel programme, while violin plots integrating box plots for key morphological traits were generated using the “ggplot2” library in R. The heritability in broad sense (H_bs_) was estimated as per Allard (1960), (H_bs_ = $\sigma_{g}^{2}/\sigma_{p}^{2}$ × 100) and were classified as low (<50%), moderate (50-80%) and high (>80%) categories. The genotypic and phenotypic coefficients of variance (GCV and PCV) were estimated as per Burton and DeVane (1953) and were categorized as low (<10%), moderate (10-15%), and high (>15%). The genetic advance estimation was carried out using the formula GA = k × H_bs_ × $\sqrt{\sigma_{p}^{2}}$ as per Allard (1960). Where k is the selection differential (k = 2.06 at 5% selection intensity), $\sigma_{p}^{2}$ is phenotypic variance, and $\sigma_{g}^{2}$ is genotypic variance. By utilizing the adjusted means of all the traits, correlations, path analysis, and principal component analysis (PCA) were estimated. The “qgraph” function in R was used to conduct Pearson’s correlation analysis for ascertaining the relationship between seed yield and other related traits that were recorded. Additionally, the “metan” package in R was employed to examine the direct and indirect contributions of various independent traits on seed yield. The associations among the traits were explored by PCA using “factoextra” and “factominer” packages.

## 3 Results

### 3.1 Performance of inter-specific derivatives for agro-morphological traits

The ANOVA for IDs during both the seasons revealed significant differences for genetic variability for all the traits. Treatments consistently showed highly significant effects, indicating strong genetic diversity. The pooled analysis showed significant effects over the years and interactions between treatments and years, emphasizing the importance of testing over the years. The alpha lattice design effectively minimized the experimental error for the evaluation of the IDs in different conditions (**Supplementary Table 1**). Moreover, the descriptive statistical measures demonstrated a wide range of variation for major agronomical parameters. The measures of descriptive statistics for important agro-morphological traits obtained across *rabi* seasons 2021-22, 2022-23 and pooled data (**Supplementary Table 2**) revealed consistent means and variability, indicating stable trait performance. The traits such as DFF, DTM and SYPP displayed reliable averages and low variability, critical for developing early maturing genotypes. Traits like NPP and SBPP remained stable across the years indicating reliable yield potential. The IDs exhibited a slight decline in yield and yield-contributing traits in 2022-23 compared to 2021-22, potentially due to higher temperatures experienced during the reproductive phases (**Supplementary Figure 1**). The data exhibited low skewness and kurtosis, signifying normal distributions, hence, facilitating statistical analysis. Overall, the results demonstrated the stability and reliability of key agro-morphological traits in chickpea across different seasons, providing valuable insights for breeding programmes aimed to enhance yield levels and overall adaptability. Further, the comparison among the IDs of crosses PBG5 × ILWC229, BGD72 × ILWC229, PBG5 × ILWC246 and BGD72 × ILWC246, displayed a wide range of variation, as evidenced by the diversity in mean, range, and CV (**Table 1**). Early flowering and maturity were observed in the *rabi* 2021-22 than in 2022-23 for all the crosses. In contrast, the IDs derived from *C. echinospermum* displayed the maximum average plant height compared to *C. reticulatum* derivatives. The IDs of crosses PBG5 × ILWC246 exhibited higher NPP and SYPP, followed by BGD72 × ILWC229, while the derivatives involving BGD72 exhibited higher HSW in comparison to derivatives involving PBG5 (**Table 1**).

**Table 1:** Range, Mean, Standard Error (SE), and Coefficient of Variation (CV) for different agro-morphological traits in chickpea inter-specific derivatives.

	*Rabi* (2021-22)			*Rabi* (2022-23)			Pooled		
Trait/Cross	Range	Mean ± SE	CV%	Range	Mean ± SE	CV%	Range	Mean ± SE	CV%
Days to 50% flowering									
PBG5 × ILWC229	96.00-108.50	102.90 ± 0.54	3.06	96.50-110.00	104.27 ± 0.68	3.77	96.25-109.25	103.65 ± 0.60	3.34
BGD72 × ILWC229	89.00-107.00	98.93 ± 0.85	4.72	92.00-110.00	100.03 ± 0.91	5.02	90.50-108.50	99.48 ± 0.86	4.77
PBG5 × ILWC246	94.00-107.00	103.25 ± 1.52	4.18	97.00-110.00	105.12 ± 1.59	4.29	95.50-108.50	104.18 ± 1.54	4.19
BGD72 × ILWC246	89.00-108.50	100.02 ± 1.11	4.86	92.00-110.00	101.44 ± 1.05	4.52	90.50-109.25	100.73 ± 1.07	4.64
Days to maturity									
PBG5 × ILWC229	144.50-153.50	149.72 ± 0.36	1.39	146.00-155.00	150.72 ± 0.37	1.41	146.00-154.25	150.28 ± 0.35	1.34
BGD72 × ILWC229	144.00-154.50	148.43 ± 0.49	1.81	145.00-155.00	150.33 ± 0.52	1.91	144.50-154.50	149.38 ± 0.48	1.78
PBG5 × ILWC246	145.00-150.50	147.00 ± 0.65	1.25	148.00-153.00	149.81 ± 0.66	1.24	146.50-151.00	148.40 ± 0.59	1.13
BGD72 × ILWC246	145.50-151.50	149.05 ± 0.42	1.24	145.50-155.00	150.47 ± 0.54	1.56	145.50-153.00	149.71 ± 0.44	1.29
Plant height (cm)									
PBG5 × ILWC229	39.49-82.49	61.85 ± 1.43	13.34	45.16-77.66	67.94 ± 1.20	10.14	42.33-80.08	64.90 ± 1.19	10.61
BGD72 × ILWC229	48.49-79.75	62.49 ± 1.43	12.58	51.50-78.00	64.50 ± 1.23	10.47	53.62-78.87	63.49 ± 1.20	10.42
PBG5 × ILWC246	46.16-74.58	65.06 ± 3.09	13.45	68.16-79.50	73.28 ± 1.25	4.82	59.24-76.87	69.17 ± 1.87	7.67
BGD72 × ILWC246	55.95-78.33	66.42 ± 1.37	9.01	58.16-78.66	69.56 ± 1.11	6.98	57.12-78.49	67.99 ± 1.19	7.65
Primary branches plant^-1^									
PBG5 × ILWC229	2.66-4.57	3.50 ± 0.08	13.12	2.83-4.99	3.88 ± 0.07	11.29	2.99-4.78	3.69 ± 0.06	10.17
BGD72 × ILWC229	2.25-4.99	3.59 ± 0.12	19.12	2.99-4.66	3.96 ± 0.07	10.71	2.87-4.58	3.78 ± 0.08	12.36
PBG5 × ILWC246	2.74-3.91	3.24 ± 0.14	12.62	3.66-4.66	4.21 ± 0.09	6.59	3.37-4.12	3.72 ± 0.09	7.43
BGD72 × ILWC246	2.41-4.75	3.68 ± 0.15	18.21	3.16-4.99	3.90 ± 0.12	13.44	2.83-4.53	3.79 ± 0.12	14.32
Secondary branches plant^-1^									
PBG5 × ILWC229	3.41-7.83	5.19 ± 0.19	22.00	3.66-7.83	5.39 ± 0.18	19.46	3.53-7.24	5.29 ± 0.17	18.97
BGD72 × ILWC229	3.25-8.41	5.46 ± 0.23	23.49	4.00-8.33	5.89 ± 0.22	21.02	3.70-7.45	5.67 ± 0.20	19.75
PBG5 × ILWC246	4.25-7.25	5.34 ± 0.32	17.35	5.33-7.50	6.41 ± 0.31	13.77	4.79-7.37	5.87 ± 0.30	14.90
BGD72 × ILWC246	4.16-7.00	5.41 ± 0.19	15.42	4.33-7.33	5.87 ± 0.17	13.00	4.24-6.62	5.64 ± 0.15	12.24
Number of pods plant^-1^									
PBG5 × ILWC229	31.08-67.66	46.27 ± 1.44	17.92	20.49-74.99	46.76 ± 1.84	17.64	25.78-71.32	46.51 ± 1.49	18.44
BGD72 × ILWC229	39.32-71.07	51.76 ± 1.80	19.14	28.83-67.50	49.25 ± 1.91	18.24	34.74-67.03	50.58 ± 1.68	18.20
PBG5 × ILWC246	41.04-62.24	52.11 ± 2.38	12.94	41.49-66.60	55.91 ± 3.24	16.42	44.72-61.20	54.01 ± 2.45	12.84
BGD72 × ILWC246	29.33-74.50	43.63 ± 2.53	19.35	28.41-65.50	47.22 ± 1.97	18.22	31.58-69.25	45.40 ± 2.14	19.54
Seeds 10 pods^-1^									
PBG5 × ILWC229	14.50-20.00	16.96 ± 0.26	9.13	15.00-20.00	18.39 ± 0.21	6.82	14.75-20.00	17.68 ± 0.21	6.89
BGD72 × ILWC229	12.50-20.00	16.73 ± 0.36	11.81	11.50-20.00	17.11 ± 0.37	12.02	12.00-20.00	16.92 ± 0.35	11.36
PBG5 × ILWC246	15.50-18.50	16.62 ± 0.39	6.77	16.00-20.00	18.12 ± 0.47	7.33	16.00-19.25	17.37 ± 0.40	6.57
BGD72 × ILWC246	15.50-20.00	17.55 ± 0.29	7.22	15.50-20.00	17.65 ± 0.31	7.67	16.00-19.25	17.60 ± 0.22	5.66
100-Seed weight (g)									
PBG5 × ILWC229	13.30-28.49	17.86 ± 0.38	12.41	13.55-28.24	18.47 ± 0.38	12.02	13.45-28.34	18.23 ± 0.38	11.97
BGD72 × ILWC229	16.68-30.56	23.61 ± 0.81	19.52	17.63-30.10	23.72 ± 0.78	18.00	17.25-29.61	23.68 ± 0.79	18.47
PBG5 × ILWC246	17.46-19.48	18.60 ± 0.24	3.73	17.53-19.63	18.57 ± 0.27	4.20	17.70-19.45	18.58 ± 0.25	3.88
BGD72 × ILWC246	16.26-26.21	18.69 ± 0.50	11.88	16.46-26.51	19.67 ± 0.56	12.59	16.53-26.39	19.28 ± 0.53	12.09
Seed yield plot^-1^ (g)									
PBG5 × ILWC229	375.50-594.50	479.19 ± 9.99	11.98	380.00-593.00	478.33 ± 9.36	11.24	380.75-593.75	478.76 ± 9.51	11.41
BGD72 × ILWC229	348.50-645.50	514.05 ± 12.97	13.82	367.50-627.00	507.86 ± 12.85	13.86	363.75-634.50	510.95 ± 12.39	13.29
PBG5 × ILWC246	464.50-574.00	515.00 ± 15.11	8.30	473.00-564.50	519.43 ± 10.67	5.81	468.75-569.25	517.21 ± 11.79	6.45
BGD72 × ILWC246	376.00-582.00	498.76 ± 12.42	10.85	385.00-577.00	497.34 ± 12.50	10.96	395.50-579.50	498.05 ± 12.33	10.79
Biological yield plot^-1^ (g)									
PBG5 × ILWC229	925.00-1525.00	1169.47 ± 24.00	11.78	900.00-1500.00	1163.63 ± 21.94	10.83	912.50-1512.50	1166.55 ± 22.79	11.22
BGD72 × ILWC229	925.00-1482.50	1242.58 ± 27.24	12.00	900.00-1475.00	1228.75 ± 26.40	11.77	912.50-1478.75	1235.66 ± 26.25	11.63
PBG5 × ILWC246	1037.50-1300.00	1205.31 ± 29.88	7.01	1050.00-1275.00	1200.00 ± 27.54	6.49	1043.75-1272.50	1202.65 ± 26.66	6.27
BGD72 × ILWC246	1025.00-1425.00	1240.26 ± 29.37	10.32	1000.00-1400.00	1232.89 ± 28.68	10.13	1012.50-1412.50	1239.21 ± 28.92	10.17
Harvest index									
PBG5 × ILWC229	29.18-45.35	41.17 ± 0.70	9.82	33.76-45.51	41.23 ± 0.61	8.63	31.77-45.44	41.20 ± 0.64	9.06
BGD72 × ILWC229	33.36-45.69	41.37 ± 0.56	7.48	33.40-45.33	41.34 ± 0.56	7.48	33.39-45.32	41.36 ± 0.53	7.04
PBG5 × ILWC246	39.39-45.46	42.74 ± 0.79	5.23	40.40-45.16	43.33 ± 0.56	3.71	40.00-45.31	43.04 ± 0.66	4.37
BGD72 × ILWC246	33.84-44.76	40.30 ± 0.70	7.65	33.92-44.38	40.23 ± 0.66	7.20	33.89-44.58	40.26 ± 0.66	7.17

**Figure 2:**
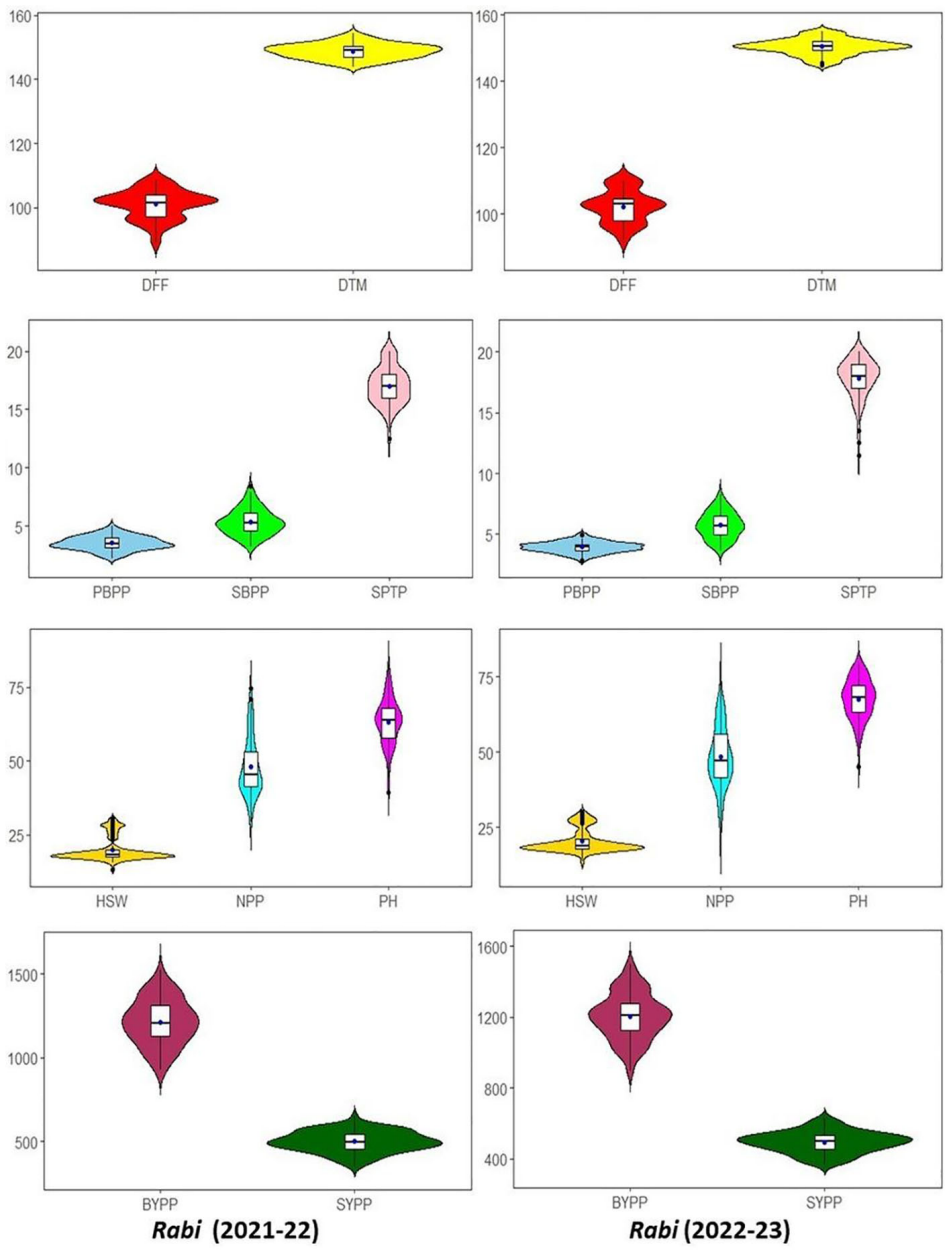
Comparative violin and boxplots of different agro-morphological traits in chickpea inter-specific derivatives for *rabi* seasons 2021-2022 and 2022-2023. (DFF-Days to 50% flowering, DTM-Days to maturity, PH-Plant height (cm), PBPP-Primary branches per plant, SBPP-Secondary branches per plant, NPP-No. of pods per plant, SPTP-Seeds per 10 pods, HSW-100 Seed weight (g), SYPP-Seed yield per plot (g), BYPP-Biological yield per plot (g)).

The assessment of variation among the IDs as determined using violin and box plots are presented in **Figure 2**. The violin plots visualize the data density and distribution with the shape indicating the concentration of data points at different values. Inside each violin plot, a box plot is integrated to provide a statistical summary. The thick black bar represents the median value, the dot within the box plot marks the interquartile range (IQR), and the thin black lines (whiskers) extend to the minimum and maximum values, excluding outliers. This combination of violin and box plots allows for a detailed comparison of central tendency, spread, and variability of the traits. For most of the traits, median values for *rabi* 2021-22 and 2022-23 were similar, suggesting the consistent performance of IDs across the seasons. In traits *viz*., DFF and DTM, seasonal variations in the spread of IDs were observed though there was overall similarity in the ranges. The PBPP, SBPP and SPTP exhibited consistent patterns across seasons, suggesting no significant seasonal variation in branching and seed producing traits. Similarly in HSW, NPP and PH traits, stable distributions were observed across the seasons. Hence, implying that these traits are relatively unaffected by seasonal changes. Lastly, BYPP and SYPP displayed symmetrical distributions with stable means across seasons, indicating that biological yield and seed yield remains consistent, although BYPP had slightly broader distribution during 2022-23 (**Figure 2**).

**Figure 3:**
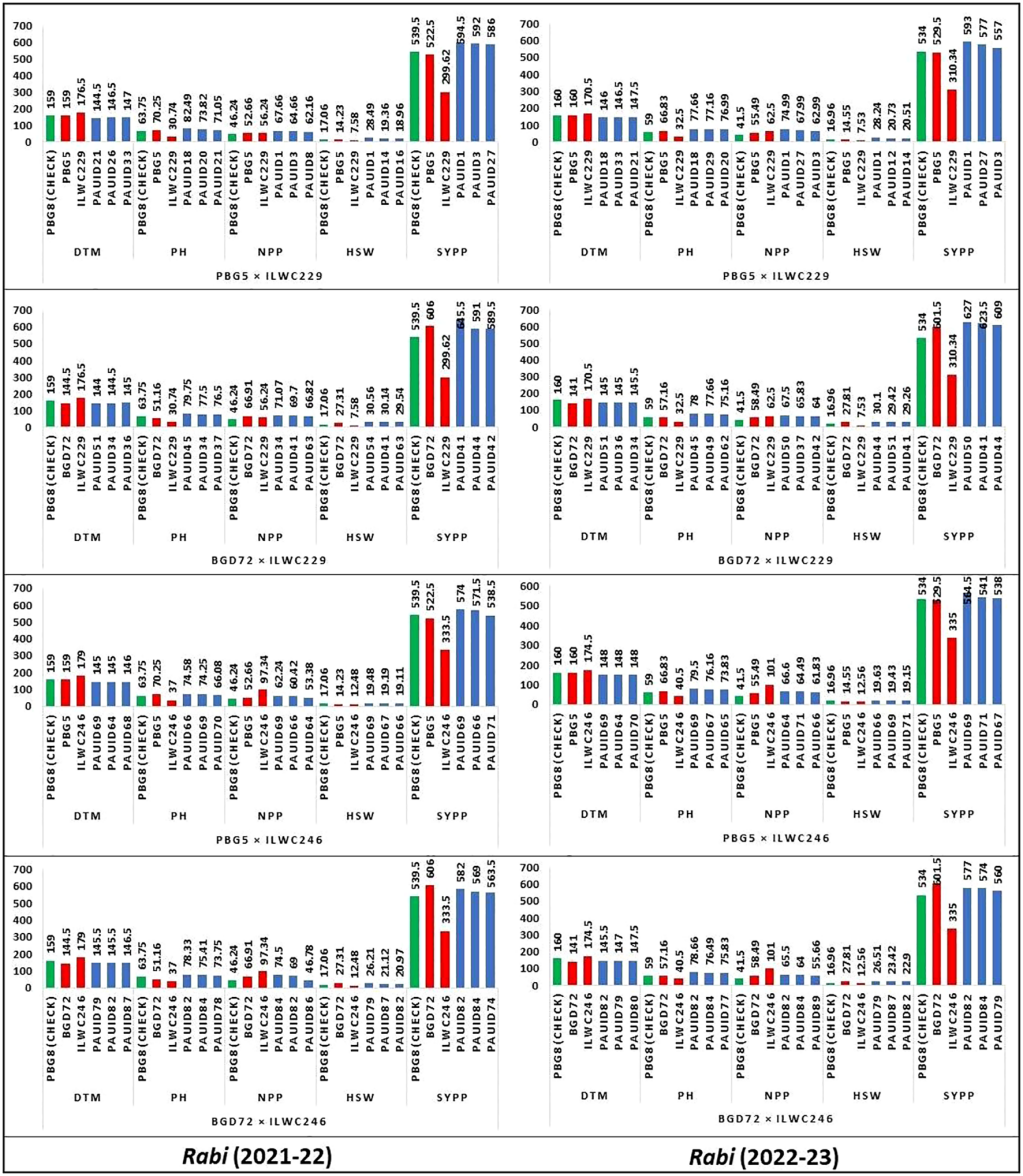
Comparative performance of chickpea inter-specific derivatives with parents, and checks for key agro-morphological traits. (DTM-Days to maturity, PH-Plant height (cm), NPP-No. of pods per plant, HSW-100 Seed weight (g), SYPP-Seed yield per plot (g)).

The comparison of chickpea traits across four crosses for two *rabi* seasons (2021-22 and 2022-23) provided valuable insights into the performance of various IDs. Each cross was analysed for five key traits namely DTM, PH, NPP, HSW and SYPP. In each cross, better-performing IDs were compared with check variety, PBG8 and parents (**Figure 3**). The data revealed that most IDs consistently outperformed the check and parents for NPP and SYPP. Hence, indicating their potential for higher productivity. Notable variations for PH and HSW were observed in IDs, with some displaying significantly better performance. Crosses with ILWC229 exhibited more variability for the traits under study compared to ILWC246. Certain IDs with higher yield levels (PAUID41, PAUID44, PAUID50 and PAUID42) could serve as potential candidates in breeding programmes aimed to improve yield and adaptability.

### 3.2 Estimation of genetic variability parameters

The analysis of genetic variability parameters for chickpea across the seasons and pooled data (**Table 2**) revealed consistently high genotypic and phenotypic variances, heritability and genetic advance for key traits such as DFF, NPP, SYPP and BYPP. The PH also exhibited high genotypic and phenotypic variances, heritability and genetic advance, though slightly lower in the pooled data compared to individual seasons. The environmental coefficient of variation was low for most of the traits, especially for highly heritable traits like DFF, PH and SYPP. There were some differences in the magnitude of genetic and environmental variances between the two seasons. For instance, the genotypic variance for PH was higher in *rabi* 2021-22 (58.04) compared to *rabi* 2022-23 (43.88), indicating a variation in the expression of this trait between seasons. Similarly, the environmental variance for traits like BYPP was higher in *rabi* 2022-23 (3849.45) compared to *rabi* 2021-22 (1064.00), reflecting the influence of different environmental conditions in each season. The genotypic coefficient of variation and phenotypic coefficient of variation also exhibit slight variations between seasons. For example, the GCV for SBPP was higher in *rabi* 2022-23 (18.46%) compared to *rabi* 2021-22 (12.29%), indicating a greater relative genetic variability in the latter season. The pooled analysis smooths out the seasonal variations and provides an average estimate of genetic and environmental parameters. This result in slightly lower heritability for some traits, like PH (70.95%) and PBPP (52.93%), compared to the individual seasons.

**Table 2:** Genetic variability parameters for various traits in chickpea across *rabi* seasons 2021-22 to 2022-23 and pooled analysis.

Trait	EV	GV	PV	ECV	GCV	PCV	H_(bs)_	GA	GAPM
*Rabi* (2021-22)									
**DFF**	2.01	19.59	21.60	1.40	4.38	4.59	90.69	8.68	8.59
**DTM**	1.37	5.28	6.65	0.78	1.54	1.73	79.37	4.21	2.83
**PH**	6.50	58.05	64.55	4.02	12.03	12.68	89.93	14.88	23.50
**PBPP**	0.06	0.32	0.38	6.85	15.91	17.32	84.36	1.06	30.10
**SBPP**	0.11	1.17	1.28	6.28	20.26	21.22	91.23	2.13	39.88
**NPP**	4.86	95.09	99.95	4.58	20.26	20.78	95.13	19.59	40.72
**SPTP**	0.50	2.41	2.91	4.18	9.14	10.05	82.71	2.91	17.13
**HSW**	0.34	16.20	16.54	2.91	20.09	20.30	97.94	8.20	40.97
**SYPP**	173.69	3725.56	3899.25	2.64	12.25	12.53	95.55	122.90	24.67
**BYPP**	1064.00	18597.28	19661.28	2.69	11.25	11.56	94.59	273.21	22.54
**HI**	0.21	11.64	11.85	1.10	8.27	8.35	98.26	6.96	16.90
*Rabi* (2022-23)									
**DFF**	0.69	23.38	24.07	0.81	4.72	4.79	97.14	9.81	9.59
**DTM**	1.04	5.47	6.51	0.67	1.55	1.69	84.01	4.41	2.93
**PH**	1.68	43.89	45.57	1.91	9.79	9.98	96.32	13.39	19.80
**PBPP**	0.02	0.19	0.21	3.66	10.97	11.57	89.93	0.84	21.43
**SBPP**	0.09	1.13	1.22	5.18	18.46	19.17	92.70	2.10	36.62
**NPP**	9.67	99.34	109.02	6.41	20.54	21.52	91.13	19.60	40.40
**SPTP**	0.17	2.67	2.84	2.29	9.19	9.47	94.15	3.26	18.37
**HSW**	0.05	14.41	14.46	1.11	18.44	18.48	99.64	7.80	37.93
**SYPP**	337.01	3373.89	3710.90	3.70	11.71	12.28	90.92	114.09	23.00
**BYPP**	3849.45	15337.08	19186.53	5.15	10.28	11.50	79.94	228.09	18.93
**HI**	1.00	9.71	10.71	2.42	7.55	7.93	90.66	6.11	14.81
Pooled									
**DFF**	1.88	20.94	22.82	1.35	4.50	4.69	91.74	9.03	8.88
**DTM**	1.99	4.60	6.59	0.94	1.43	1.71	69.79	3.68	2.46
**PH**	15.99	39.06	55.05	6.10	9.54	11.33	70.95	10.84	15.56
**PBPP**	0.14	0.15	0.29	9.91	10.51	14.45	52.93	0.59	15.75
**SBPP**	0.37	0.88	1.25	11.01	16.88	20.16	70.15	1.61	29.13
**NPP**	28.43	76.06	104.49	11.03	18.05	21.16	72.79	15.32	31.73
**SPTP**	0.96	1.92	2.88	5.64	7.96	9.76	66.57	2.32	13.38
**HSW**	0.62	14.88	15.50	3.88	18.99	19.39	95.98	7.78	38.34
**SYPP**	439.07	3366.00	3805.07	4.21	11.67	12.41	88.46	112.40	22.61
**BYPP**	2340.38	17083.52	19423.90	4.00	10.81	11.53	87.95	252.50	20.90
**HI**	1.35	9.93	11.28	2.81	7.64	8.14	88.02	6.08	14.76

EV, Environmental Variance; GV, Genotypic Variance; PV, Phenotypic Variance; ECV, Environmental Coefficient of Variation; GCV, Genotypic Coefficient of Variation; PCV, Phenotypic Coefficient of Variation; H(bs), Heritability in broad sense; GA, Genetic advance; GAPM, Genetic advance as percentage of mean; DFF, Days to 50% flowering; DTM, Days to maturity; PH, Plant height; PBPP, Primary branches per plant; SBPP, Secondary branches per plant; NPP, No. of pods per plant; SPTP, Seeds per 10 pods; HSW, 100 Seed weight; SYPP, Seed yield per plot; BYPP, Biological yield per plot; HI, Harvest index.

### 3.3 Correlations and path analysis

**Figure 4:**
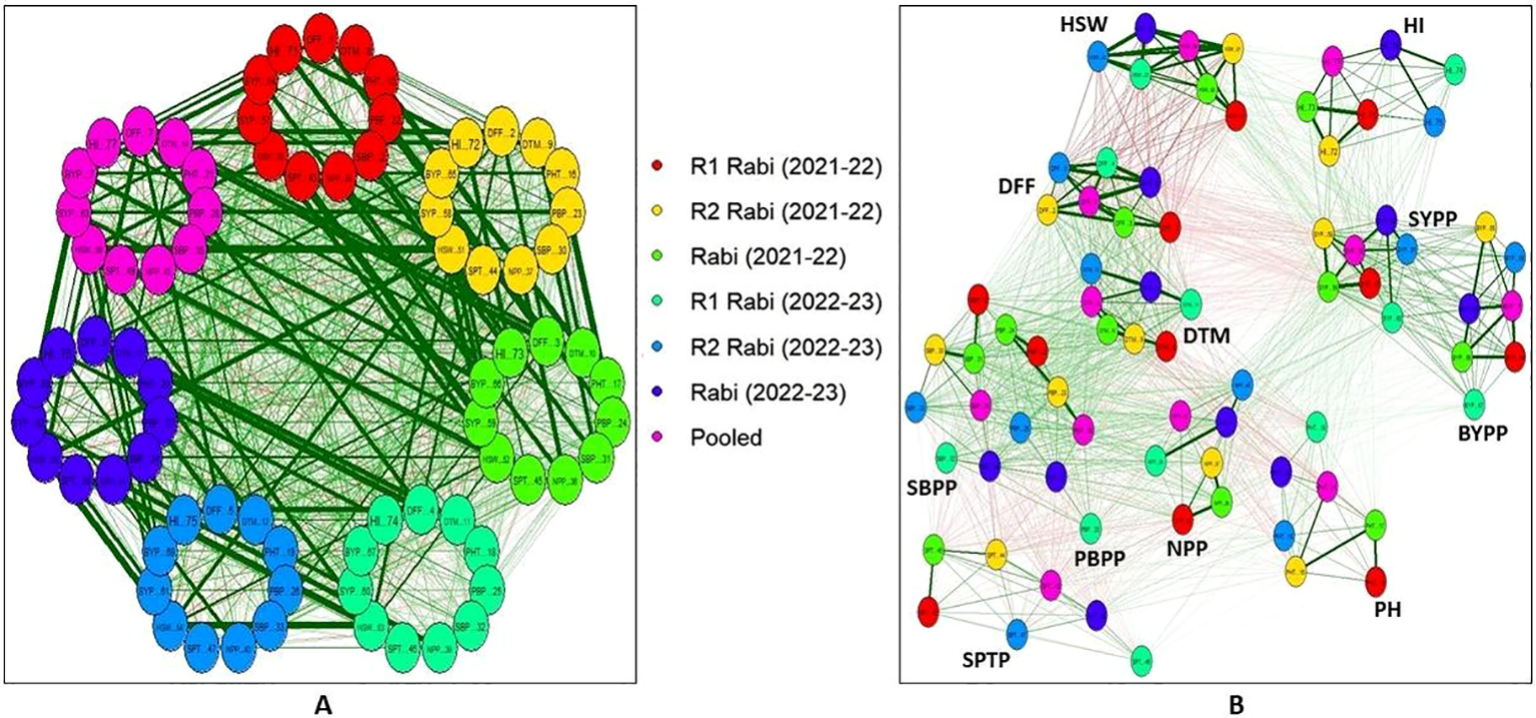
Pearson’s correlation networks in chickpea inter-specific derivatives: **(A)** Inter-trait relationships across seasons **(B)** Seasonal consistency of trait correlations (Green edges indicate positive correlations, while red edges indicate negative correlations. The thickness and saturation of these edges reflect the strength of the absolute correlation). (R1-Replication 1, R2-Replication 2, *rabi* (2021-22) is R1+R2 of 2021-22, *rabi* (2022-22) is R1+R2 of 2022-23). (DFF-Days to 50% flowering, DTM-Days to maturity, PH-Plant height (cm), PBPP-Primary branches per plant, SBPP-Secondary branches per plant, NPP-No. of pods per plant, SPTP-Seeds per 10 pods, HSW-100 Seed weight (g), SYPP-Seed yield per plot (g), BYPP-Biological yield per plot (g) and HI-Harvest index).

Visualizing correlation matrices as networks (**Figure 4**), where variables are represented as nodes and the correlations between them as connecting edges, reveals the important relationships among chickpea traits. Using this approach, the network analysis of chickpea traits across different *rabi* seasons and pooled data revealed strong positive correlations amongst key traits, such as NPP with SYPP and BYPP with HI. SYPP was also having positive correlation with HSW, PH, SBPP and PBPP. Additionally, positive correlations were observed between DFF and DTM while BYPP with NPP, SBPP, HSW, PH and PBPP. Interestingly, DFF and DTM were negatively correlated with SYPP. All these correlations were consistent across both seasons and the pooled analysis. The analysis also revealed distinct clusters of inter-related traits, such as SYPP, BYPP and HI and another cluster comprising of DFF, DTM and HSW.

**Figure 5:**
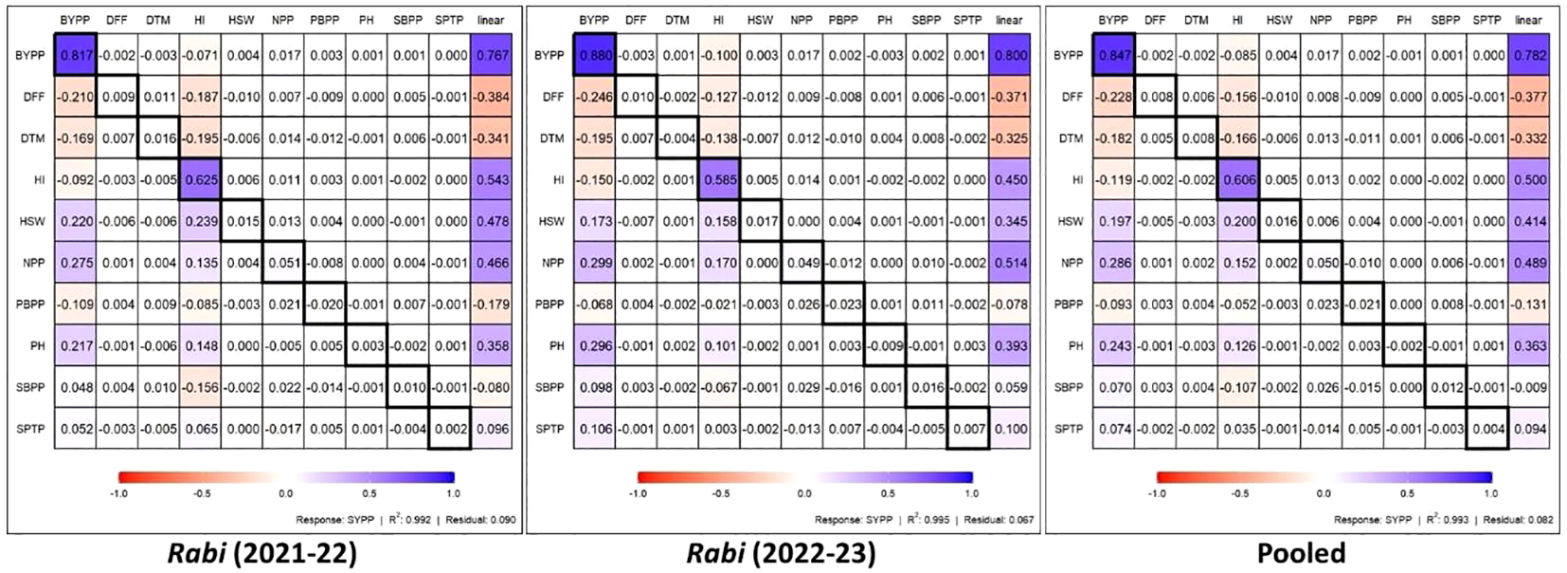
Direct (diagonal) and indirect contributions of various traits to seed yield across the seasons and pooled analysis. (DFF-Days to 50% flowering, DTM-Days to maturity, PH-Plant height (cm), PBPP-Primary branches per plant, SBPP-Secondary branches per plant, NPP-No. of pods per plant, SPTP-Seeds per 10 pods, HSW-100 Seed weight (g), SYPP (linear)-Seed yield per plot (g), BYPP-Biological yield per plot (g) and HI-Harvest index).

The path analysis results for chickpea traits across different *rabi* seasons and pooled data (**Figure 5**) revealed the direct and indirect effects of the studied traits on SYPP. Out of all the 11 traits, BYPP showed consistent strong direct positive effect on SYPP across season 2021-22, season 2022-23 and pooled data, with 0.817, 0.880 and 0.847, path coefficients, respectively. The HI also demonstrated a significant positive direct effect on SYPP across all datasets, with coefficients of 0.625, 0.585 and 0.606 for season 2021-22, season 2022-23 and pooled data. Traits such as HSW, NPP and PH exhibited moderate positive indirect effects on SYPP via BYPP and HI across the years and pooled analysis. The negative indirect effects of DFF and DTM on SYPP were observed to be more pronounced during *rabi* 2022-23. The lower residual values across different *rabi* seasons and pooled data (0.090, 0.067 and 0.082, respectively) indicate that majority of the yield attributes were included in the path analysis.

### 3.4 Principal component analysis

**Figure 6:**
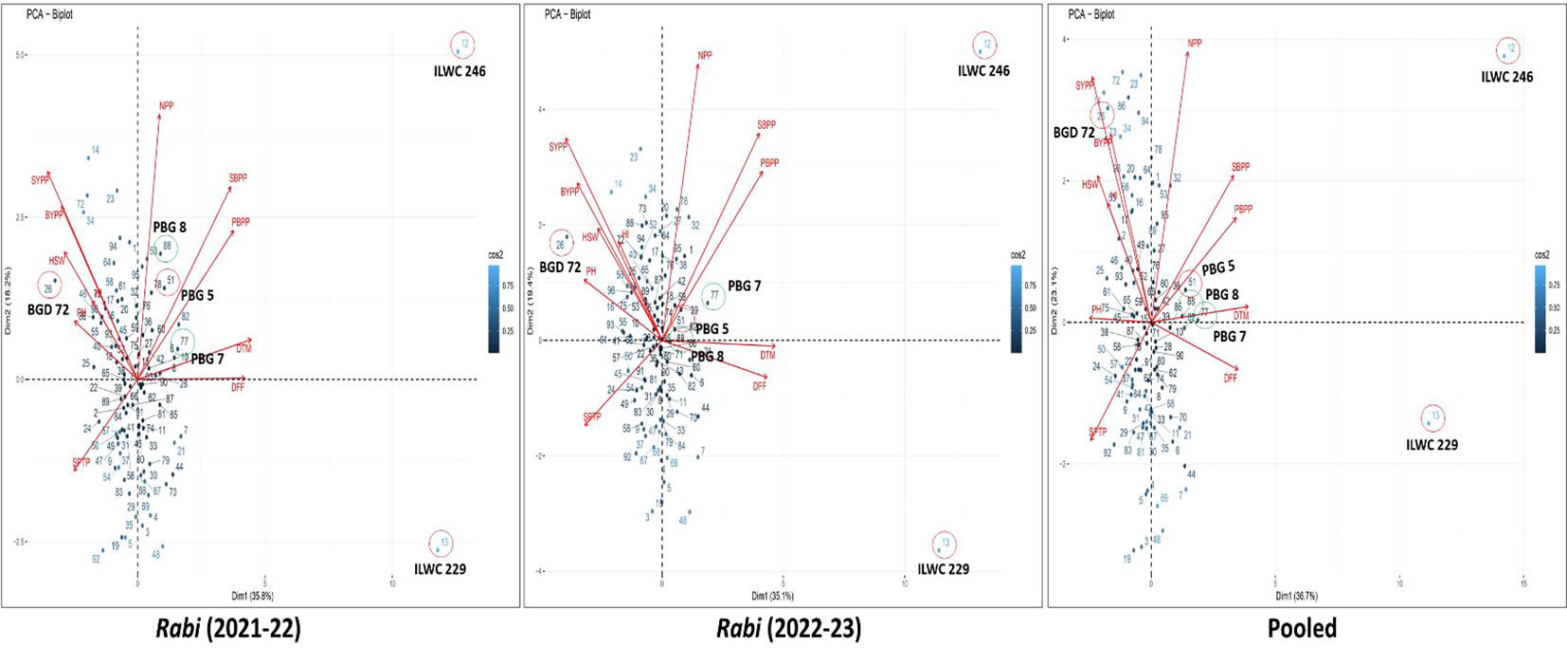
PCA biplots depicting yield relationships in inter-specific derivatives of chickpea across the seasons and pooled analysis.

The PCA biplots for *rabi* 2021-22, 2022-23 and pooled data (**Figure 6**) revealed both similarities and differences in the contributions of traits to SYPP. Across all datasets, traits such as NPP, SYPP, BYPP and HSW consistently had long vectors. In contrast, DFF and DTM vectors were in similar directions but away from yield traits, highlighting an inverse relationship with seed yield. The biplot of pooled data explained a higher percentage of the total variance (59.8%) compared to the individual *rabi* seasons (54% for 2021-22 and 54.5% for 2022-23), indicating a more comprehensive capture of trait variability on combining seasons. The distribution of genotypes across all biplots had similar patterns thereby reinforcing the consistent associations between traits and genotypes. The strong positive associations of NPP, SYPP, BYPP and HSW highlights the direct influence on yield, while negative associations for DFF and DTM with yield traits. According to the biplot analysis, the ID 94 (PAUID44) followed by 20 (PAUID54) and 72 (PAUID41) were found to be superior for traits such as SYPP, BYPP and HSW across the seasons and the pooled analysis.

### 3.5 Screening against diseases

**Figure 7:**
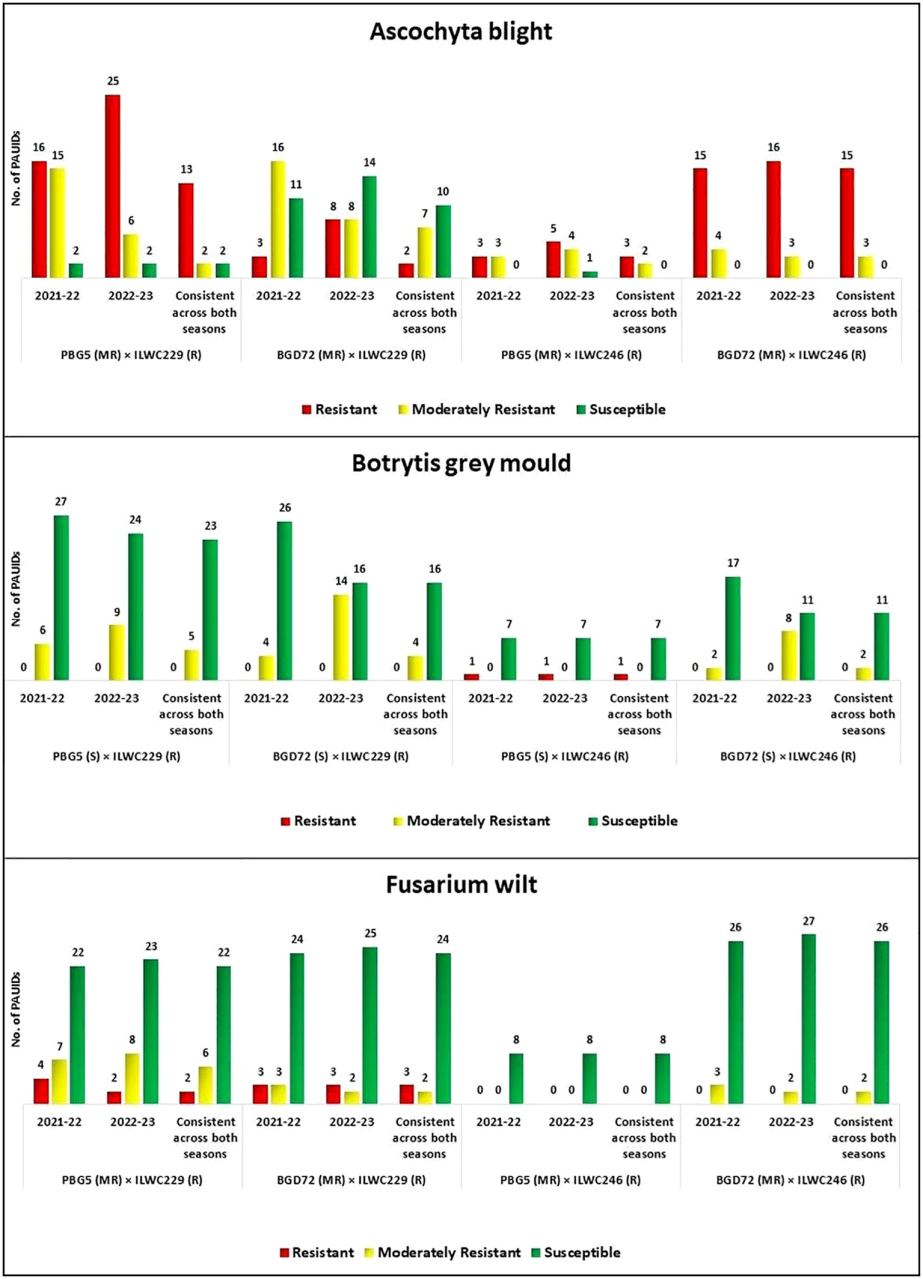
Performance of chickpea inter-specific derivatives against Ascochyta blight, botrytis grey mould, and fusarium wilt [R (Resistant), MR (Moderately Resistant) and S (Susceptible)].

The four parent lines had varying levels of disease resistance: PBG5 and BGD72 were moderately resistant to AB and FW but susceptible to BGM. In contrast, ILWC229 and ILWC246 were resistant to all three diseases (**Figure 7**).

#### 3.5.1 Ascochyta blight

A total of 33 derivatives (13 from PBG5 × ILWC229, 2 from BGD72 × ILWC229, 3 from PBG5 × ILWC246 and 15 from BGD72 × ILWC246 cross) showed resistance against AB, while 14 derivatives (2 from PBG5 × ILWC229, 7 from BGD72 × ILWC229, 2 from PBG5 × ILWC246 and 3 from BGD72 × ILWC246 cross) exhibited moderate resistance across both seasons (**Figure 7**) consistently. The remaining derivatives were either susceptible or highly susceptible to the disease.

#### 3.5.2 Botrytis grey mould

The screening results indicated that across both seasons (**Figure 7**) out of 90, only one ID (PAUID69) of cross PBG5 × ILWC246 gave resistant disease reaction while moderate level of resistance was observed in 11 derivatives (5 from PBG5 × ILWC229, 4 from BGD72 × ILWC229 and 2 from BGD72 × ILWC246 cross).

#### 3.5.3 Fusarium wilt

The screening of chickpea IDs against FW revealed that five derivatives from crosses PBG5 × ILWC229 (2 IDs) and BGD72 × ILWC229 (3 IDs) exhibited resistant disease reactions against the pathogen. Likewise, a moderate level of resistance was observed in 10 derivatives (6 of PBG5 × ILWC229, 2 of BGD72 × ILWC229, and 2 of BGD72 × ILWC246 cross) consistently across the seasons (**Figure 7**). The remaining derivatives were either susceptible or highly susceptible to FW.

### 3.6 Crude protein content

**Figure 8:**
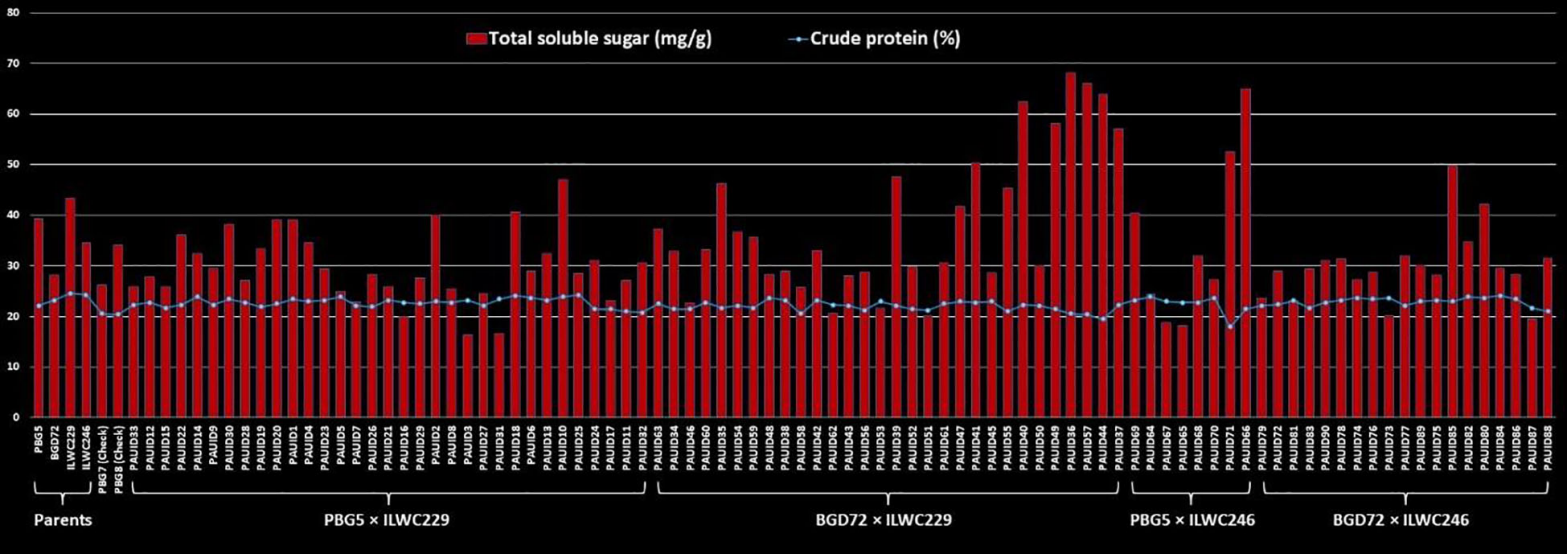
Comparison of crude protein (%) and total soluble sugar (mg/g) contents in inter-specific derivatives of chickpea.

The range of estimated crude protein content for 90 IDs varied significantly across the different crosses (17.93% to 24.28%), indicating the presence of sufficient genetic diversity and potential for selection among the derivatives. The cross PBG5 × ILWC246 exhibited the widest range for crude protein content (17.93% to 23.84%), followed by BGD72 × ILWC246 (21.00% to 24.06%), PBG5 × ILWC229 (20.78% to 24.28%) and BGD72 × ILWC229 (19.46% to 23.62%) (**Table 3**). A slight variation for mean crude protein content was observed among the crosses, BGD72 × ILWC246 (22.87%), PBG5 × ILWC229 (22.71%), PBG5 × ILWC246 (22.31%) and BGD72 × ILWC229 (21.94%) (**Figure 8**).

**Table 3:** Estimates of crude protein and total soluble sugar content in chickpea inter-specific derivatives.

Trait/Cross	Crude protein (%)		Total soluble sugars (mg/g)	
	Range	Mean ± SE	Range	Mean ± SE
**PBG5 × ILWC229**	20.78-24.28	22.71 ± 0.15	16.25-47.00	30.34 ± 1.17
**BGD72 × ILWC229**	19.46-23.62	21.94 ± 0.17	20.06-68.06	38.64 ± 2.64
**PBG5 × ILWC246**	17.93-23.84	22.31 ± 0.67	18.12-65.00	34.79 ± 5.92
**BGD72 × ILWC246**	21.00-24.06	22.87 ± 0.19	19.43-49.75	29.95 ± 1.60

### 3.7 Total soluble sugars

The crosses in this study exhibited a wide range of total soluble sugar content, reflecting the genetic potential for improving the trait. The cross BGD72 × ILWC229 exhibited the broadest range of total soluble sugar content, from 20.06 mg/g to 68.06 mg/g. This was followed by PBG5 × ILWC246 (18.12 mg/g to 65.00 mg/g), BGD72 × ILWC246 (19.43 mg/g to 49.75 mg/g) and PBG5 × ILWC229 (16.25 mg/g to 47.00 mg/g) (**Table 3**). Among these crosses, BGD72 × ILWC229 had the highest average total soluble sugar content (38.64 mg/g), followed by PBG5 × ILWC246 (34.79 mg/g), PBG5 × ILWC229 (30.34 mg/g) and BGD72 × ILWC246 (29.95 mg/g) (**Figure 8**).

## 4 Discussion

In crop improvement programmes, there is a mounting trend of pre-breeding and genetic enhancement activities by involving wild relatives to discover novel genes and alleles, thereby expanding the genetic diversity in released cultivars (Singh et al., 2015). The habitual use of the same breeding parents has led to the narrow genetic base of the major pulse crops including chickpea. It has been reported by Kumar et al. (2003) that about 41% of hybridized chickpea varieties have PB7 as a common ancestor. Enhancement of genetic diversity by incorporating beneficial characteristics from wild relatives into cultivated gene pools through wide hybridization is common in cereals, pulses, oilseeds and fiber crop species. In chickpea, this strategy has proven successful in introducing traits related to productivity, resistance to both biotic and abiotic stresses, and in widening the genetic diversity (Bhavyasree et al., 2018a, Bhavyasree et al., 2018b; Kushwah et al., 2021a; Salaria et al., 2023). A breeding attempt of distant hybridization was made by crossing cultivated varieties (PBG5 and BGD72) with *C. reticulatum* (ILWC229) and *C. echinospermum* (ILWC246) as male parents (Singh et al., 2018) with the aim to transfer productivity traits and resistance to major chickpea diseases. Numerous studies have reported successful inter-specific hybridization events in cultivated chickpea. Some of the easily crossable annual wild species include *C. echinospermum* (Singh et al., 2018, 2022a, 2022b, 2022c, Sari et al., 2022) and *C. reticulatum* (Singh et al., 2005; Adak et al., 2017; Bhavyasree et al., 2018a, Bhavyasree et al., 2018b; Singh et al., 2018; Sari et al., 2022).

The present experimental findings demonstrated significant variation among the derivatives derived from hybridization of *C. arietinum* with the wild annual *Cicer* species (*C. reticulatum* and *C. echinospermum*), as evidenced by the analysis of variance, range, mean and coefficient of variation for key agro-morphological traits which was also supported by earlier studies (Bhavyasree et al., 2018a, b; Kushwah et al., 2021b, c).

Furthermore, the determination of genotypic and phenotypic coefficients of variation with boxplot analyses revealed consistent variations for traits like PH, NPP and SYPP. High heritability coupled with substantial genetic advance was observed for most of the traits across seasons. This indicated the probable stabilization of additive gene effects (Singh et al., 2022b) that can ease the selection of genetic materials with the desirable traits. Studies by Kushwah et al. (2021b) and Singh et al. (2022c) emphasizing the significance of genetic variance for yield and its related characteristics, corroborated the results of present study. Assessments of derivatives for NPP and SYPP align with the findings of Singh et al. (2005) and Pal et al. (2005). Similar findings were also observed by Singh and Ocampo (1997) and Singh et al. (2015) for yield and its related characteristics in chickpea. Higher variability for seed yield among the inter-specific derivatives of chickpea was also observed earlier by Kanaka et al. (2007); Sidramappa et al. (2008) and Farshadfar and Farshadfar (2008).

Traits with high genotypic and phenotypic coefficients of variation demonstrated considerable genetic variability, which is advantageous for breeding programmes. The low environmental variance for most traits indicated that environmental factors have a limited impact on trait expression. The close match between genotypic variance and phenotypic variance further supports that the observed variation is primarily genetic with least influence of environment (Johnson et al., 1955). Overall, these results demonstrated that the traits studied were largely controlled by genetic factors and have significant potential for improvement through selection. High heritability and genetic advance values for key traits like HSW, NPP and SYPP suggested that these traits can be effectively targeted in breeding programmes to enhance chickpea productivity and adaptability.

Correlation studies are important for understanding how different variables impact the genetic makeup of a crop. The mutual relationship between two variables is determined by degree of correlation. Seed yield is affected by several other traits and by analyzing the relationships, seed yield can be improved through selection based on those traits. In the present study, the traits like BYPP, HI, NPP, HSW and PH were identified as important yield components which should be taken into consideration during the selection programme for yield improvement in chickpea. Johnson et al. (2015) also identified biological yield and pods per plant as important contributing traits towards high yield in chickpea. These findings emphasized the importance of these traits in breeding programmes. Overall, the dense network of interactions underscores the complexity of trait relationships. Thus, suggesting that a holistic approach in breeding programmes can lead to substantial improvements in chickpea productivity, adaptability and resilience to varying environmental conditions. The negative indirect effects of days to flowering and maturity on seed yield were more pronounced during *rabi* 2022-23, suggesting that environmental conditions had more significant impact on these traits. The lower residual values across the seasons and for pooled analysis indicated that important yield contributing traits were appropriately included in the study. These insights may provide a strategic roadmap to the breeders for the development of high yielding chickpea cultivars by targeting key traits in selection programmes.

The observed decline in yield and yield-contributing traits in 2022-23, compared to 2021-22, suggested that higher temperatures during the reproductive phases adversely affected the plants’ productivity. This indicated the sensitivity of these traits to temperature fluctuations, highlighting the need for developing heat-tolerant varieties. Previous studies by Gaur et al. (2007); Paul et al. (2018) and Kushwah et al. (2021b), Kushwah et al. (2021c) have indicated that heat-stress environments significantly affect most morphological traits. Reduced seed yield during heat stress conditions could be attributed to low pollen viability (Kushwah et al., 2021b). Additionally, pollen sterility has been identified as a major factor contributing to poor pod setting under pre-anthesis high-temperature stress (Devasirvatham et al., 2010).

Across all datasets, traits such as pods per plant, seed yield, biological yield and seed weight consistently had long vectors, indicated their strong contributions to the principal components and positive correlations with each other. This suggested that these traits are key drivers of variability and are essential for enhancing yield as also reported by Kushwah et al. (2021b). On the other hand, days to flowering and maturity suggested that there is a need to keep a desired balance of the negative association between early maturity and yield. The stability of these patterns across seasons enhance the reliability of these traits for selection in breeding programmes. Overall, the PCA biplots provided a strategic roadmap for developing high-yielding, adaptable chickpea cultivars by focusing on the traits studied.

Effective screening and identification of resistant sources are crucial for developing cultivars that can withstand the major pathogens. In this study, screening of chickpea inter-specific derivatives against AB, BGM and FW revealed significantly promising results. Earlier, *C. reticulatum* and *C. echinospermum* have been reported to harbour high level of resistance against AB (Stamigna et al., 1998). In a study by Singh et al. (2014), ILWC229 (*C. reticulatum*) was reported to have high level of resistance to AB and ILWC246 (*C*. *echinospermum*) resistance to AB and BGM. Based on findings of these studies, ILWC229 and ILWC246 were used as donors for AB and BGM to generate inter-specific derivatives which were screened consecutively for two seasons. In all, 33 and 14 IDs against AB, 1 and 11 IDs against BGM and 5 and 10 IDs against FW were found resistant and moderately resistant, respectively (**Supplementary Table 3**). This indicated presence of considerable repository for inter-species defence mechanism governed by novel genes in the wild *Cicer* species. Promising inter-specific derivatives having resistance to AB and BGM were also identified earlier by various workers in chickpea (Singh et al., 2005; Kaur et al., 2013; Bhavyasree et al., 2018a; Kushwah et al., 2021a; Salaria et al., 2023) indicated the potential of wild *Cicer* species for generating derivatives resistant to economically important diseases. A high yielding BGM resistant chickpea cultivar, PBG8, was developed through introgression of BGM resistance from *C. judaicum* by Singh et al. (2022) strongly supported that the promising resistant derivatives identified in the present study could be further deployed for the development of disease-resistant cultivars. The findings of our study demonstrated that wild *Cicer* species harbour valuable and untapped variations for productivity traits and resistance to major chickpea diseases. By utilising these promising lines in breeding programmes, robust chickpea cultivars resistant to AB, BGM and FW could be developed, thereby ensuring more resilient and productive crops.

The analysis of protein and total soluble sugars content provided a valuable insight to the nutritional composition in derivatives under study. The crude protein content in chickpea is of significant interest due to its implications for their nutritional value as chickpeas are known for high protein content, making them an essential source of plant-based protein in both vegetarian and non-vegetarian diets worldwide (Singh, 1985; Jukanti et al., 2012). The total soluble sugars play a crucial role in determining the taste, flavour, palatability and nutritional quality contribute towards consumer’s acceptance of chickpea-based products. The significant differences in levels of crude protein and total soluble sugars in the derivatives provided an insight to the amount of genetic diversity created from crosses involving wild *Cicer* species *C. reticulatum* and *C. echinospermum.* The promising derivatives possessing higher protein and soluble sugar contents can further be used in breeding programmes to enhance the nutritional value and flavour within cultivated germplasm. Furthermore, the relationship between total soluble sugars and crude protein content warrants in-depth consideration in chickpea breeding. As by balancing these two nutritional components along with optimal agronomic performance the overall farmers’ preference, consumers’ acceptance and market competitiveness of chickpea cultivars could be enhanced. By building on this work, we can contribute significantly to sustainable agriculture and food security initiatives hence, ensuring the development of chickpea cultivars to meet the evolving needs of both producers and consumers.

## 5 Conclusions

Significant variability was observed for most of the agro-morphological traits, nutritional parameters and resistance to major chickpea diseases. The inter-specific derivatives exhibited greater stability, higher yield potential and enhanced level of resistance to major diseases namely Ascochyta blight, Botrytis grey mould and Fusarium wilt. These findings accentuate the potential of wild species as a vital resource for broadening the genetic base of chickpea. The consistent performance (adaptability and resilience) of these derivatives across seasons make them prime candidates to be utilized in breeding programmes for developing robust, high-yielding nutritionally-rich disease resistant cultivars. Further, the identified derivatives carrying desired traits, such as resistance to major diseases and higher nutritional contents could serve as valuable genetic resources for germplasm enhancement. The identified promising derivatives can be instrumental in future breeding programmes for ensuring the continued improvement and adaptation of chickpea cultivars to meet the growing demand and to tackle future agricultural challenges for sustainable and resilient chickpea crop.

## Data Availability

The original contributions presented in the study are included in the article/**Supplementary Material**. Further inquiries can be directed to the corresponding author.
