# Supplementary material for: Evaluation and identification of advanced inter-specific derivatives from crosses of Cicer arietinum with C. reticulatum and C. echinospermum for agro-morphological, quality traits and disease resistance
Source: Front Plant Sci. 2024 Sep 27;15:1461280. doi: 10.3389/fpls.2024.1461280 (PMC11466825; doi:10.3389/fpls.2024.1461280)
Supplement: Supplementary file 1 [file Table1.docx]

Supplementary Material

**Evaluation and identification of advanced inter-specific derivatives from crosses of *Cicer arietinum* with *C. reticulatum* and *C. echinospermum* for agro-morphological, quality traits and disease resistance**

**Vadithya Amool Singh^1^, Inderjit Singh^1^, Upasana Rani^1^, Sreya Venadan^1^, Rajdeep Jajoriya^1^, Mohar Singh^2^, Harpreet Kaur Oberoi^1^, Sarvjeet Singh^1^, Chellapilla Bharadwaj^3^, Shayla Bindra^1*^**

^1^Department of Plant Breeding and Genetics, Punjab Agricultural University, Ludhiana, India

^2^Indian Council of Agricultural Research (ICAR)-National Bureau of Plant Genetic Resources Regional Station, Shimla, India

^3^Division of Genetics, ICAR- Indian Agricultural Research Institute, New Delhi, India

*** Correspondence:**Corresponding Author
shaylabindra@pau.edu

ORCID ID: 0000-0002-8155-6451

## Supplementary Figures


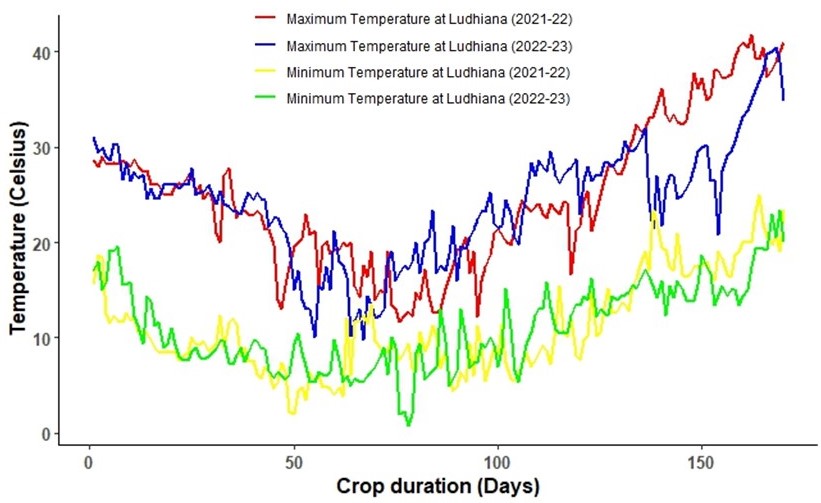


**Supplementary Fig. 1** The daily maximum and minimum temperatures for 2021-22 and 2022-23 during the crop period at Ludhiana.
