# Supplementary material for: Evaluation and identification of advanced inter-specific derivatives from crosses of Cicer arietinum with C. reticulatum and C. echinospermum for agro-morphological, quality traits and disease resistance
Source: Front Plant Sci. 2024 Sep 27;15:1461280. doi: 10.3389/fpls.2024.1461280 (PMC11466825; doi:10.3389/fpls.2024.1461280)
Supplement: Supplementary file 2 [file Table2.docx]

Supplementary Material

**Evaluation and identification of advanced inter-specific derivatives from crosses of *Cicer arietinum* with *C. reticulatum* and *C. echinospermum* for agro-morphological, quality traits and disease resistance**

**Vadithya Amool Singh^1^, Inderjit Singh^1^, Upasana Rani^1^, Sreya Venadan^1^, Rajdeep Jajoriya^1^, Mohar Singh^2^, Harpreet Kaur Oberoi^1^, Sarvjeet Singh^1^, Chellapilla Bharadwaj^3^, Shayla Bindra^1*^**

^1^Department of Plant Breeding and Genetics, Punjab Agricultural University, Ludhiana, India

^2^Indian Council of Agricultural Research (ICAR)-National Bureau of Plant Genetic Resources Regional Station, Shimla, India

^3^Division of Genetics, ICAR- Indian Agricultural Research Institute, New Delhi, India

*** Correspondence:**Corresponding Author
shaylabindra@pau.edu

ORCID ID: 0000-0002-8155-6451

## Supplementary Tables

**Supplementary Table 1.** ANOVA for yield and related traits of chickpea inter-specific derivatives.

| **Source** | **DF** | **DFF** | **DTM** | **PH** | **PBPP** | **SBPP** | **NPP** | **SPTP** | **HSW** | **SYPP** | **BYPP** | **HI** |
| --- | --- | --- | --- | --- | --- | --- | --- | --- | --- | --- | --- | --- |
| ***Rabi* (2021-22)** | | | | | | | | | | | | |
| **Replications** | 1 | 33.33* | 10.08* | 482.82* | 5.04* | 6.79* | 55.18* | 28.52* | 5.49* | 1170.2* | 5156* | 0.21 |
| **Treatment (unadj)** | 95 | 87.47* | 51.24* | 155.14* | 1.90* | 4.61* | 242.11* | 7.27* | 37.94* | 9510.2* | 42799* | 28.14* |
| **Block/Replication** | 14 | 1.95 | 1.22 | 7.11 | 0.03 | 0.11 | 6.72 | 0.69 | 0.46 | 163.1 | 1314 | 0.22 |
| **Residuals** | 81 | 1.97 | 1.56 | 6.29 | 0.06 | 0.11 | 4.45 | 0.46 | 0.29 | 164.3 | 954 | 0.19 |
| ***Rabi* (2022-23)** | | | | | | | | | | | | |
| **Replications** | 1 | 9.63* | 18.75* | 87.29* | 3.22* | 6.11* | 41.27* | 3.25 | 0.04 | 1091.0* | 8868 | 0.01 |
| **Treatment (unadj)** | 95 | 87.16* | 38.47* | 128.06* | 1.44* | 4.66* | 261.54* | 7.95* | 35.08* | 8191.5* | 38593* | 21.07* |
| **Block/Replication** | 14 | 1.10 | 0.93 | 38.62 | 0.51 | 2.08 | 17.38 | 2.39 | 0.05 | 575.5 | 6064 | 0.84 |
| **Residuals** | 81 | 0.59 | 1.00 | 23.70 | 0.29 | 1.27 | 8.61 | 2.57 | 0.04 | 285.4 | 3332 | 1.02 |
| **Pooled** | | | | | | | | | | | | |
| **Treatment** | 95 | 171.53* | 85.20* | 244.32* | 3.00* | 8.37* | 435.62* | 13.09* | 71.63* | 16872.5* | 79335* | 45.78* |
| **Year** | 1 | 141.37* | 156.31* | 1603.08* | 12.19* | 14.30 | 16.11 | 62.56* | 26.64* | 147.0 | 5767 | 3.00* |
| **Treatment : Year** | 95 | 3.10* | 4.52* | 38.88* | 0.34* | 0.90* | 68.04* | 2.13* | 1.39* | 829.2* | 2057 | 3.43* |
| **Year : Replication** | 2 | 21.48* | 14.41* | 285.06* | 4.13* | 6.45* | 48.23* | 15.88* | 2.77* | 1130.6* | 7012* | 0.11 |
| **Year : Rep : Block** | 28 | 1.52 | 1.07 | 22.87* | 0.27* | 1.10* | 12.06* | 1.54 | 0.26* | 369.3* | 3689* | 0.53 |
| **Residuals** | 162 | 1.28 | 1.28 | 15.00 | 0.17 | 0.69 | 6.54 | 1.52 | 0.17 | 224.9 | 2143 | 0.60 |

**p* = 0.05

DF-Degrees of freedom, DFF-Days to 50% flowering, DTM-Days to maturity, PH-Plant height, PBPP-Primary branches per plant, SBPP-Secondary branches per plant, NPP-No. of pods per plant, SPTP-Seeds per 10 pods, HSW-100 Seed weight, SYPP-Seed yield per plot, BYPP-Biological yield per plot, HI-Harvest index

**Supplementary Table. 2** Summary of descriptive statistical measures for important agro-morphological traits.

| **Trait** | **Min** | **Max** | **Mean** | **Std. Error** | **Std. Deviation** | **Skewness** | **Kurtosis** |
| --- | --- | --- | --- | --- | --- | --- | --- |
| ***Rabi* (2021-22)** | | | | | | | |
| **DFF** | 89.00 | 108.50 | 101.00 | 0.47 | 4.51 | -0.51 | 0.006 |
| **DTM** | 144.00 | 154.50 | 148.91 | 0.24 | 2.35 | 0.03 | -0.52 |
| **PH** | 39.49 | 82.49 | 63.31 | 0.82 | 7.82 | -0.23 | 0.56 |
| **PBPP** | 2.25 | 4.99 | 3.55 | 0.06 | 0.59 | 0.06 | -0.35 |
| **SBPP** | 3.25 | 8.41 | 5.34 | 0.11 | 1.10 | 0.48 | -0.06 |
| **NPP** | 29.33 | 74.50 | 48.06 | 1.03 | 9.82 | 0.71 | -0.007 |
| **SPTP** | 12.50 | 20.00 | 16.98 | 0.17 | 1.63 | -0.03 | -0.09 |
| **HSW** | 13.30 | 30.56 | 20.02 | 0.42 | 4.04 | 1.33 | 0.53 |
| **SYPP** | 348.50 | 645.50 | 498.12 | 6.50 | 61.74 | -0.15 | -0.40 |
| **BYPP** | 925.00 | 1525.00 | 1211.97 | 14.57 | 138.30 | -0.02 | -0.58 |
| **HI** | 29.18 | 45.69 | 41.19 | 0.36 | 3.42 | -1.02 | 0.83 |
| ***Rabi* (2022-23)** | | | | | | | |
| **DFF** | 92.00 | 110.00 | 102.33 | 0.51 | 4.87 | -0.04 | -0.66 |
| **DTM** | 145.00 | 155.00 | 150.46 | 0.25 | 2.41 | -0.21 | -0.17 |
| **PH** | 45.16 | 79.50 | 67.61 | 0.70 | 6.68 | -0.56 | 0.45 |
| **PBPP** | 2.83 | 4.99 | 3.94 | 0.04 | 0.44 | -0.04 | -0.16 |
| **SBPP** | 3.66 | 8.33 | 5.75 | 0.11 | 1.08 | 0.16 | -0.65 |
| **NPP** | 20.49 | 74.99 | 48.50 | 1.07 | 10.20 | 0.09 | 0.08 |
| **SPTP** | 11.50 | 20.00 | 17.78 | 0.17 | 1.66 | -1.29 | 2.35 |
| **HSW** | 13.55 | 30.10 | 20.48 | 0.40 | 3.81 | 1.14 | 0.14 |
| **SYPP** | 367.50 | 627.00 | 495.84 | 6.27 | 59.51 | -0.09 | -0.34 |
| **BYPP** | 900.00 | 1500.00 | 1203.19 | 13.80 | 130.98 | 0.01 | -0.30 |
| **HI** | 33.40 | 45.51 | 41.24 | 0.33 | 3.19 | -0.80 | -0.12 |
| **Pooled** | | | | | | | |
| **DFF** | 90.50 | 109.25 | 101.69 | 0.48 | 4.62 | -0.27 | -0.40 |
| **DTM** | 144.50 | 154.50 | 149.69 | 0.23 | 2.25 | -0.10 | -0.20 |
| **PH** | 42.33 | 80.08 | 65.46 | 0.69 | 6.56 | -0.39 | 0.85 |
| **PBPP** | 2.83 | 4.78 | 3.74 | 0.04 | 0.43 | -0.04 | -0.47 |
| **SBPP** | 3.53 | 7.45 | 5.54 | 0.10 | 0.98 | 0.05 | -0.84 |
| **NPP** | 25.78 | 71.32 | 48.30 | 0.96 | 9.11 | 0.53 | -0.02 |
| **SPTP** | 12.00 | 20.00 | 17.38 | 0.15 | 1.46 | -0.88 | 1.77 |
| **HSW** | 13.45 | 29.61 | 20.30 | 0.40 | 3.87 | 1.23 | 0.28 |
| **SYPP** | 363.75 | 634.50 | 496.98 | 6.21 | 58.95 | -0.17 | -0.35 |
| **BYPP** | 912.50 | 1512.50 | 1208.13 | 14.01 | 132.92 | -0.03 | -0.42 |
| **HI** | 31.77 | 45.44 | 41.22 | 0.33 | 3.20 | -0.92 | 0.28 |

DFF-Days to 50% flowering, DTM-Days to maturity, PH-Plant height, PBPP-Primary branches per plant, SBPP-Secondary branches per plant, NPP-No. of pods per plant, SPTP-Seeds per 10 pods, HSW-100 Seed weight, SYPP-Seed yield per plot, BYPP-Biological yield per plot, HI-Harvest index

**Supplementary Table. 3** List of IDs showing resistance and moderate level of resistance to major diseases.

| **Disease/Disease reaction** | **No of IDs** | **Name of IDs** |
| --- | --- | --- |
| **Ascochyta blight** | | |
| Resistant | 33 | PAUID5, PAUID6, PAUID7, PAUID8, PAUID9, PAUID16, PAUID19, PAUID21, PAUID22, PAUID23, PAUID24, PAUID25, PAUID33, PAUID53, PAUID57, PAUID64, PAUID65, PAUID66, PAUID72, PAUID73, PAUID74, PAUID75, PAUID76, PAUID77, PAUID78, PAUID80, PAUID81, PAUID84, PAUID85, PAUID86, PAUID87, PAUID89, PAUID90 |
| Moderately Resistant | 14 | PAUID11, PAUID32, PAUID36, PAUID40, PAUID43, PAUID45, PAUID46, PAUID60, PAUID62, PAUID67, PAUID69, PAUID79, PAUID82, PAUID88 |
| **Botrytis grey mould** | | |
| Resistant | 1 | PAUID69 |
| Moderately Resistant | 11 | PAUID4, PAUID5, PAUID7, PAUID15, PAUID22, PAUID40, PAUID58, PAUID61, PAUID62, PAUID76, PAUID84 |
| **Fusarium wilt** | | |
| Resistant | 5 | PAUID18, PAUID33, PAUID37, PAUID55, PAUID62 |
| Moderately Resistant | 10 | PAUID3, PAUID12, PAUID19, PAUID20, PAUID24, PAUID31, PAUID43, PAUID45, PAUID83, PAUID84 |

**Supplementary Table. 4** Details about the 96 inter-specific derivatives and principal component analysis (PCA) ID.

| **S. No** | **Derivative ID** | **CROSS (Parentage)** | **PCA ID** |
| --- | --- | --- | --- |
| 1 | PAUID63 | CROSS-2 (BGD72 × ILWC229) | 1 |
| 2 | PAUID79 | CROSS-4 (BGD72 × ILWC246) | 2 |
| 3 | PAUID33 | CROSS-1 (PBG5 × ILWC229) | 3 |
| 4 | PAUID12 | CROSS-1 (PBG5 × ILWC229) | 4 |
| 5 | PAUID72 | CROSS-4 (BGD72 × ILWC246) | 5 |
| 6 | PAUID15 | CROSS-1 (PBG5 × ILWC229) | 6 |
| 7 | PAUID22 | CROSS-1 (PBG5 × ILWC229) | 7 |
| 8 | PAUID81 | CROSS-4 (BGD72 × ILWC246) | 8 |
| 9 | PAUID14 | CROSS-1 (PBG5 × ILWC229) | 9 |
| 10 | PAUID9 | CROSS-1 (PBG5 × ILWC229) | 10 |
| 11 | PAUID30 | CROSS-1 (PBG5 × ILWC229) | 11 |
| 12 | ILWC246 | Parent | ILWC246 |
| 13 | ILWC229 | Parent | ILWC229 |
| 14 | PAUID34 | CROSS-2 (BGD72 × ILWC229) | 14 |
| 15 | PAUID28 | CROSS-1 (PBG5 × ILWC229) | 15 |
| 16 | PAUID46 | CROSS-2 (BGD72 × ILWC229) | 16 |
| 17 | PAUID69 | CROSS-3 (PBG5 × ILWC246) | 17 |
| 18 | PAUID60 | CROSS-2 (BGD72 × ILWC229) | 18 |
| 19 | PAUID35 | CROSS-2 (BGD72 × ILWC229) | 19 |
| 20 | PAUID54 | CROSS-2 (BGD72 × ILWC229) | 20 |
| 21 | PAUID19 | CROSS-1 (PBG5 × ILWC229) | 21 |
| 22 | PAUID20 | CROSS-1 (PBG5 × ILWC229) | 22 |
| 23 | PAUID1 | CROSS-1 (PBG5 × ILWC229) | 23 |
| 24 | PAUID59 | CROSS-2 (BGD72 × ILWC229) | 24 |
| 25 | PAUID48 | CROSS-2 (BGD72 × ILWC229) | 25 |
| 26 | BGD72 | Parent | BGD72 |
| 27 | PAUID64 | CROSS-3 (PBG5 × ILWC246) | 27 |
| 28 | PAUID4 | CROSS-1 (PBG5 × ILWC229) | 28 |
| 29 | PAUID23 | CROSS-1 (PBG5 × ILWC229) | 29 |
| 30 | PAUID5 | CROSS-1 (PBG5 × ILWC229) | 30 |
| 31 | PAUID83 | CROSS-4 (BGD72 × ILWC246) | 31 |
| 32 | PAUID38 | CROSS-2 (BGD72 × ILWC229) | 32 |
| 33 | PAUID58 | CROSS-2 (BGD72 × ILWC229) | 33 |
| 34 | PAUID42 | CROSS-2 (BGD72 × ILWC229) | 34 |
| 35 | PAUID7 | CROSS-1 (PBG5 × ILWC229) | 35 |
| 36 | PAUID90 | CROSS-4 (BGD72 × ILWC246) | 36 |
| 37 | PAUID26 | CROSS-1 (PBG5 × ILWC229) | 37 |
| 38 | PAUID21 | CROSS-1 (PBG5 × ILWC229) | 38 |
| 39 | PAUID78 | CROSS-4 (BGD72 × ILWC246) | 39 |
| 40 | PAUID62 | CROSS-2 (BGD72 × ILWC229) | 40 |
| 41 | PAUID16 | CROSS-1 (PBG5 × ILWC229) | 41 |
| 42 | PAUID29 | CROSS-1 (PBG5 × ILWC229) | 42 |
| 43 | PAUID43 | CROSS-2 (BGD72 × ILWC229) | 43 |
| 44 | PAUID2 | CROSS-1 (PBG5 × ILWC229) | 44 |
| 45 | PAUID56 | CROSS-2 (BGD72 × ILWC229) | 45 |
| 46 | PAUID74 | CROSS-4 (BGD72 × ILWC246) | 46 |
| 47 | PAUID53 | CROSS-2 (BGD72 × ILWC229) | 47 |
| 48 | PAUID39 | CROSS-2 (BGD72 × ILWC229) | 48 |
| 49 | PAUID8 | CROSS-1 (PBG5 × ILWC229) | 49 |
| 50 | PAUID76 | CROSS-4 (BGD72 × ILWC246) | 50 |
| 51 | PBG5 | Parent | PBG5 |
| 52 | PAUID67 | CROSS-3 (PBG5 × ILWC246) | 52 |
| 53 | PAUID3 | CROSS-1 (PBG5 × ILWC229) | 53 |
| 54 | PAUID65 | CROSS-3 (PBG5 × ILWC246) | 54 |
| 55 | PAUID52 | CROSS-2 (BGD72 × ILWC229) | 55 |
| 56 | PAUID27 | CROSS-1 (PBG5 × ILWC229) | 56 |
| 57 | PAUID68 | CROSS-3 (PBG5 × ILWC246) | 57 |
| 58 | PAUID31 | CROSS-1 (PBG5 × ILWC229) | 58 |
| 59 | PAUID73 | CROSS-4 (BGD72 × ILWC246) | 59 |
| 60 | PAUID51 | CROSS-2 (BGD72 × ILWC229) | 60 |
| 61 | PAUID18 | CROSS-1 (PBG5 × ILWC229) | 61 |
| 62 | PAUID70 | CROSS-3 (PBG5 × ILWC246) | 62 |
| 63 | PAUID77 | CROSS-4 (BGD72 × ILWC246) | 63 |
| 64 | PAUID61 | CROSS-2 (BGD72 × ILWC229) | 64 |
| 65 | PAUID47 | CROSS-2 (BGD72 × ILWC229) | 65 |
| 66 | PAUID89 | CROSS-4 (BGD72 × ILWC246) | 66 |
| 67 | PAUID6 | CROSS-1 (PBG5 × ILWC229) | 67 |
| 68 | PAUID13 | CROSS-1 (PBG5 × ILWC229) | 68 |
| 69 | PAUID75 | CROSS-4 (BGD72 × ILWC246) | 69 |
| 70 | PAUID10 | CROSS-1 (PBG5 × ILWC229) | 70 |
| 71 | PAUID85 | CROSS-4 (BGD72 × ILWC246) | 71 |
| 72 | PAUID41 | CROSS-2 (BGD72 × ILWC229) | 72 |
| 73 | PAUID82 | CROSS-4 (BGD72 × ILWC246) | 73 |
| 74 | PAUID25 | CROSS-1 (PBG5 × ILWC229) | 74 |
| 75 | PAUID80 | CROSS-4 (BGD72 × ILWC246) | 75 |
| 76 | PAUID45 | CROSS-2 (BGD72 × ILWC229) | 76 |
| 77 | PBG7 | Check | PBG7 |
| 78 | PAUID84 | CROSS-4 (BGD72 × ILWC246) | 78 |
| 79 | PAUID55 | CROSS-2 (BGD72 × ILWC229) | 79 |
| 80 | PAUID24 | CROSS-1 (PBG5 × ILWC229) | 80 |
| 81 | PAUID86 | CROSS-4 (BGD72 × ILWC246) | 81 |
| 82 | PAUID40 | CROSS-2 (BGD72 × ILWC229) | 82 |
| 83 | PAUID87 | CROSS-4 (BGD72 × ILWC246) | 83 |
| 84 | PAUID17 | CROSS-1 (PBG5 × ILWC229) | 84 |
| 85 | PAUID11 | CROSS-1 (PBG5 × ILWC229) | 85 |
| 86 | PAUID50 | CROSS-2 (BGD72 × ILWC229) | 86 |
| 87 | PAUID49 | CROSS-2 (BGD72 × ILWC229) | 87 |
| 88 | PBG8 | Check | PBG8 |
| 89 | PAUID71 | CROSS-3 (PBG5 × ILWC246) | 89 |
| 90 | PAUID32 | CROSS-1 (PBG5 × ILWC229) | 90 |
| 91 | PAUID88 | CROSS-4 (BGD72 × ILWC246) | 91 |
| 92 | PAUID36 | CROSS-2 (BGD72 × ILWC229) | 92 |
| 93 | PAUID57 | CROSS-2 (BGD72 × ILWC229) | 93 |
| 94 | PAUID44 | CROSS-2 (BGD72 × ILWC229) | 94 |
| 95 | PAUID66 | CROSS-3 (PBG5 × ILWC246) | 95 |
| 96 | PAUID37 | CROSS-2 (BGD72 × ILWC229) | 96 |
